# Bumblebees land rapidly and robustly using a sophisticated modular flight control strategy

**DOI:** 10.1016/j.isci.2021.102407

**Published:** 2021-04-24

**Authors:** Pulkit Goyal, Antoine Cribellier, Guido C.H.E. de Croon, Martin J. Lankheet, Johan L. van Leeuwen, Remco P.M. Pieters, Florian T. Muijres

**Affiliations:** 1 Experimental Zoology Group, Wageningen University and Research, 6708 WD Wageningen, the Netherlands; 2 Control and Simulation, Faculty of Aerospace Engineering, Delft University of Technology, 2629 HS Delft, the Netherlands

**Keywords:** Biological Sciences, Zoology, Ethology, Mathematical Biosciences

## Abstract

When approaching a landing surface, many flying animals use visual feedback to control their landing. Here, we studied how foraging bumblebees (*Bombus terrestris*) use radial optic expansion cues to control in-flight decelerations during landing. By analyzing the flight dynamics of 4,672 landing maneuvers, we showed that landing bumblebees exhibit a series of deceleration bouts, unlike landing honeybees that continuously decelerate. During each bout, the bumblebee keeps its relative rate of optical expansion constant, and from one bout to the next, the bumblebee tends to shift to a higher, constant relative rate of expansion. This modular landing strategy is relatively fast compared to the strategy described for honeybees and results in approach dynamics that is strikingly similar to that of pigeons and hummingbirds. The here discovered modular landing strategy of bumblebees helps explaining why these important pollinators in nature and horticulture can forage effectively in challenging conditions; moreover, it has potential for bio-inspired landing strategies in flying robots.

## Highlights

• A custom-developed analysis method reveals how bumblebees control their landing
• Landing bumblebees stepwise regulate the set point of optical expansion rate
• Bumblebees land robustly in various challenging environmental conditions

## Graphical abstract

**Figure:**
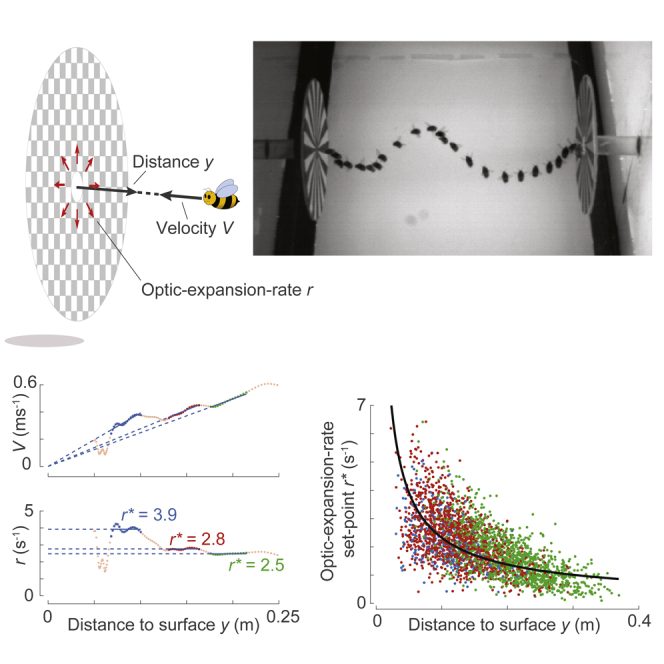

## Introduction

Landing is essential for all flying animals, and successful landings require precise control of flight momentum to perform soft touchdown. This is particularly relevant for foraging animals that use flight to routinely collect food. For example, bumblebees can perform more than 1000 landing maneuvers on flowers per hour (Heinrich, 1979). For each landing, the animal uses its sensory-motor system to control deceleration in such a manner that its flight speed reduces to near zero at touchdown, thereby maximizing landing success and minimizing the risk of impact injuries (Foster and Cartar, 2011).

Many flying animals, including birds and insects, use visual motion cues to control approach speed during landings (Lee et al., 1991; Lee et al., 1993; Van Breugel and Dickinson, 2012; Baird et al., 2013; Chang et al., 2016). The animal's motion relative to the landing surface generates a radially expanding optic flow field, in which various features in the image appear to move radially outward from the center of expansion (Gibson, 1955; Edwards and Ibbotson, 2007). Flying animals can use this rate of optical expansion along with the retinal size of an object (Wagner, 1982) or angular position of features in the visual field (Baird et al., 2013) to compute the “relative rate of expansion (*r*)” or its inverse, the instantaneous “time to contact” (τ = 1/*r*, referred to as parameter *tau* in literature) (Lee, 1976; Sun and Frost, 1998; Lee et al., 2009; Balebail et al., 2019). The relative rate of expansion provides information about the ego-motion of the animal and equals the ratio between approach speed *V* and distance from the landing surface *y* (*r* = *V*/*y*); instantaneous time-to-contact equals the time until contact with the landing surface, should the animal continue to fly at its current flight speed (τ = *y*/*V*). The animals can use this relative rate of expansion (or time to contact) to gradually reduce their flight speed when approaching the landing surface and touch down at near-zero speed (Lee et al., 1991; Lee et al., 1993, 2009; Baird et al., 2013).

**Figure 1:**
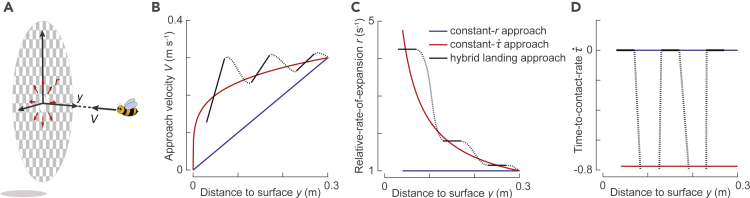
Illustration of landing strategies described in honeybees (blue) (Baird et al., 2013), birds (red) (Lee et al., 1991; Lee et al., 1993), and bumblebees as observed in this study (black). (A) An animal that approaches a vertical landing platform along its axial direction experiences a relative optical expansion rate *r* as symbolized by the red arrows. At time *t*, the animal is at distance *y* from the object, has an approach flight velocity *V*, experiences a relative rate of expansion of *r* = *V*/*y*, and has an instantaneous time-to-contact τ = *y*/*V*. (B–D) The variation with distance from the landing surface of (B) approach velocity *V*, (C) relative rate of expansion *r*, and (D) time-to-contact rate ($\dot{\tau }$ = dτ/dt) for the constant-*r* landing approach observed in honeybees (blue) (Baird et al., 2013), the constant-$\dot{\tau }$ landing approach of birds (red) (Lee et al., 1991; Lee et al., 1993), and the here-observed hybrid landing approach of bumblebees (black). The hybrid landing approach consists of constant-*r* segments (solid lines), separated by transition phases (dotted curves). All results, and particularly the transition phases, are of idealized cases. Because birds and insects differ in size, there are large differences in distances and velocities between these landing strategies. For comparative purposes, we here show idealized versions of the three landing strategies with speeds and distances typical for bumblebees and honeybees, as all landings start at 0.3 m distance from the landing surface with an approach velocity of 0.3 m s^−1^.

Birds and insects decelerate during landing in different ways (Figure 1). Honeybees (*Apis mellifera ligustica*) have been shown to approach a landing surface (up until ∼7 cm distance from the surface) by keeping the relative rate of expansion constant at a particular set point (Baird et al., 2013). By doing so, their approach speed decreases linearly with distance to the landing surface (Figure 1B). Fruit flies (*Drosophila melanogaster*) and bumblebees (*Bombus impatiens*) have been suggested to use similar strategies (Van Breugel and Dickinson, 2012; Baird et al., 2013; Chang et al., 2016). Pigeons (*Columba livia*) and hummingbirds (*Colibri coruscans*), on the other hand, approach a landing surface by keeping the derivative of instantaneous time to contact constant (Figures 1B–1D, at a negative value as per sign convention in Figure 1A) (Lee et al., 1991; Lee et al., 1993). This derivative of “time to contact” ($\dot{\text{τ}}$) is hereafter referred to as “time-to-contact rate” and defines how fast the animal decreases its time to contact, or increases its relative rate of expansion, during the landing maneuver (Figure 1C). Compared to honeybees, the avian landing strategy results in higher approach flight speeds, and hence faster landings (Figure 1B). From here on, we refer to the avian landing strategy as the constant-$\dot{\text{τ}}$ strategy and the honeybee landing strategy as the constant-*r* strategy. Note that the constant-*r* strategy is a special case of the constant-$\dot{\text{τ}}$ strategy whereby $\dot{\text{τ}}$ is maintained at a value of zero ($\dot{\text{τ}}$ = 0).

Here, we study the landing maneuver dynamics of bumblebees (*Bombus terrestris*). Bumblebees are important pollinators in both nature and horticulture (Fontaine et al., 2006; Velthuis and van Doorn, 2006; Joar Hegland and Totland, 2008) owing to their ability to forage in a wide range of environmental conditions including relatively low temperatures (Corbet et al., 1993) and limited light conditions such as during twilight hours (Reber et al., 2015, 2016). Moreover, foraging bumblebees are efficient pollinators as they are able to visit more than 1000 flowers per hour (Heinrich, 1979). During such fast foraging actions, bumblebees tend to rapidly move from flower to flower in a single flower patch, followed by longer distance flights between patches. As a result, the average distance traveled between flowers in a fresh clover field is approximately 0.33 m (Heinrich, 1979).

**Figure 2:**
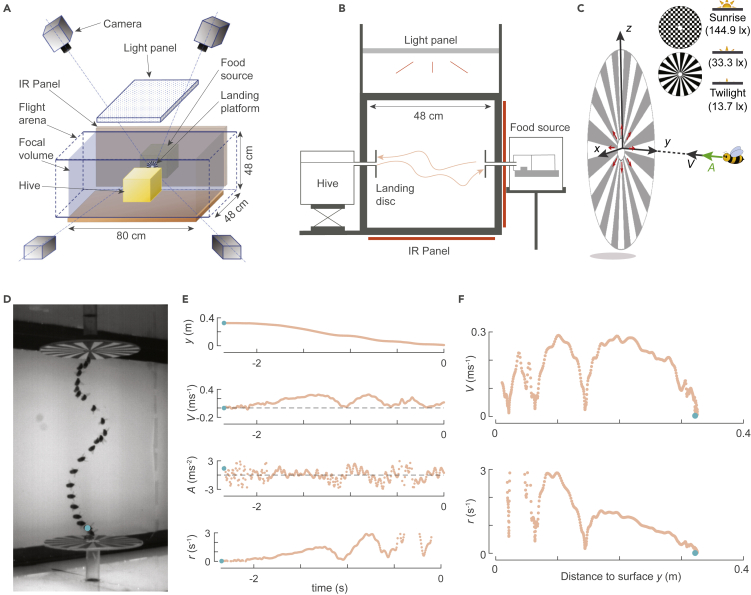
Experimental setup, definitions of the landing kinematics parameters, and temporal dynamics of a typical landing maneuver. (A and B) The experimental setup consists of a flight arena with a four-camera high-speed videography system for tracking flying bumblebees, two vertical landing platforms connected to a hive and food source (outside the arena), and a LED light panel for varying the light conditions. (C) The landing kinematics are described in a Cartesian coordinate system with its origin at the center of the landing platform, the *z* axis vertically up, and the *y* axis aligned along the axis of the disc and pointing into the flight arena. For each landing, we determined the temporal dynamics of approach distance *y*, velocity *V* = −*v*_*y*_, and acceleration *A* = −*a*_*y*_ along the *y* axis. The different landing patterns and light conditions used in this study are also shown. (D–F) Flight dynamics of a bumblebee taking off and landing on a spoke landing platform; in all panels, the blue circle denotes the start of the flight sequence. (D) Photomontage from a downward-facing camera of the landing maneuver, at a time interval of ∼0.1 s. (E) Temporal dynamics of the kinematics parameters (*y*, *V*, *A*) and the optical relative rate of expansion *r* = *V*/*y*, where time *t* = 0 s at touchdown. (F) The variation of *V* and *r* with perpendicular distance from the platform *y*.

To reproduce these foraging conditions, we trained bumblebees to routinely fly back and forth between two vertical landing platforms, one connected to their colony and the other to a food source (Figures 2A and 2B). We placed the landing platforms 0.34 m apart, which is similar to an average distance of 0.33 m traveled by bumblebees between landings when foraging on a fresh flower patch (Heinrich, 1979). The setup was placed in a large flight arena (Figures 2A and 2B), allowing the bumblebees to also exhibit the larger distance flights that resemble those between flower patches (Heinrich, 1979).

Using machine vision techniques, we then tracked 10,005 landing maneuvers of bumblebees. This data set consists of 2792 landings performed directly after taking off from the opposite platform or the ground, and 7213 landings following free flight. Moreover, to test how environmental conditions affect these landings, we varied the light intensity in three steps from twilight to sunrise conditions, and used two landing platforms with relatively low and high optical expansion information (Figure 2C).

We used two approaches to analyze the temporal deceleration dynamics of the landings. First, we analyzed how the average of multiple landing approaches varied among treatments (light condition and landing platform type) and type of landing maneuvers (landing after takeoff and from free flight). This analysis strategy is similar to the one used previously to study the landing dynamics of bumblebees (*B. impatiens*) and honeybees (Baird et al., 2013; Chang et al., 2016). Second, we analyzed how the flight dynamics of individual landing maneuvers vary among the treatments and between landings after takeoff and from free flight. Hereafter, we refer to the former and the latter as the *average-per-treatment* and *per-track* analysis methods, respectively.

Our average-per-treatment analysis provides similar results as reported previously (Baird et al., 2013; Chang et al., 2016), showing that, on average, landing bumblebees decelerate linearly with reducing distance, in all tested conditions. This suggests that bumblebees use a constant-*r* landing strategy during both landings after takeoff and from free flight. In contrast, our per-track analysis shows that “individual bumblebees” do not do so, as they exhibit short intervals of deceleration at different set points of the relative rate of expansion (Figure 1). During each set point, bumblebees keep their relative rate of expansion constant, and they increase their set point value as they reach closer to the surface. In fact, this increase in set points of relative rate of expansion with decreasing distance from the landing surface is governed on an average by a constant-$\dot{\text{τ}}$ law, with $\dot{\text{τ}}$ values similar to those of birds. Thus, on average, landing bumblebees approximate the landing strategies of birds by adjusting their constant-*r* set point in discrete steps as they approach the landing surface. Hence, this modular landing strategy of bumblebees can best be characterized as a hybrid between the constant-*r* and constant-$\dot{\text{τ}}$ strategies described for honeybees and birds, respectively.

## Results

We trained a hive of bumblebees (*B. terrestris*) to forage for food in a flight arena equipped with a real-time automatic machine-vision-based three-dimensional insect tracking system (Straw et al., 2011) (Figure 2A). We placed a food source and hive on either side of the flight arena and connected them to two vertical landing platforms (0.18 m diameter). To collect food, the foraging bumblebees flew between the landing platforms and walked through the small aperture (0.02 m diameter) in the middle of the platform to access either the hive or the food source (Figure 2B).

During the experiments, we used landing platforms with either checkerboard or spoke patterns, as they provide a high and low amount of optical expansion flow information, respectively (Figure 2C). In addition, we varied the light intensity in the setup in three levels ranging from twilight to sunrise, referred to as low (13.7 lx), medium (33.3 lx), and high (144.9 lx) light conditions. Bumblebees continued to forage in all light conditions, allowing us to test how the landing strategy varied throughout the natural variation of challenging light conditions experienced by foraging bumblebees (Figure 2C). Light and platform conditions were systematically varied such that all combinations were tested (Table S1).

We placed the two landing platforms 0.34 m apart from each other, such that it resembles the 0.33 m average distance traveled between flowers by bumblebees foraging in a fresh clover patch (Heinrich, 1979). The flight arena (3 × 0.48 × 0.48 m; length × width × height) was large enough to capture not only landings directly after takeoff from the other platform but also landings from free flight (Figure S1). These landings represent those exhibited by bumblebees when traveling between flower patches or when traveling between the hive and foraging site.

**Figure 3:**
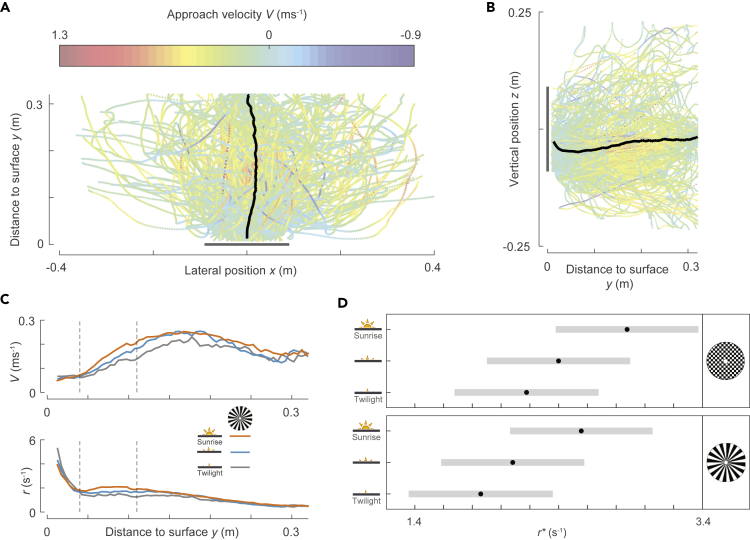
The average flight kinematics of bumblebees approaching a landing platform. (A and B) Top and side views of every 35^th^ flight trajectory of all 10,005 recorded landing maneuvers (*n* = 288 tracks), color-coded with approach velocity *V*. The black curve shows the mean trajectory of all 10,005 recorded maneuvers, and the landing platform is shown in gray. (C and D) The average approach kinematics of bumblebees. (C) The approach velocity *V* and relative rate of expansion *r* versus perpendicular distance from the platform *y* for bumblebees approaching a spoke pattern for landings initiated from free flight in low, medium, and high light conditions (in gray, blue, and orange, respectively). The *y* segment (0.04 m ≤*y*≤ 0.11 m) for which the data are used to find the mean relative rate of expansion *r*^∗^ is highlighted in dashed black lines. (D) *r*^∗^ as predicted by the linear mixed-effects model for the three tested light conditions and two landing patterns for landings of free-flying bumblebees (see methods). The mean relative rate of expansion increases with increase in light intensity but did not differ significantly between the two tested landing platforms (Table S2). Black dots depict estimated means, and gray bars are 95% confidence intervals. (See also Figures S2 and S3 and Table S2).

We used the insect tracking system to determine the three-dimensional spatial-temporal dynamics of body location in 10,005 flight maneuvers of bumblebees approaching the landing platforms. Out of 10,005 landing approaches, 2792 landings followed after a takeoff from the ground or the opposite platform (Figures 2D–2F, Video S1) and 7213 landings occurred after free flight (Figures 3A and 3B). Irrespective of how bumblebees initiated their landing, most approach flights consisted of both acceleration and deceleration phases (Figure 2E). We hereafter focused only on the deceleration phases as we aimed to find out how bumblebees slowed down during their landing maneuver. For the landings following takeoff, the flight speed at the start of the landing maneuver was *U*_start_ = 0.11 [0.04, 0.24] m/s (median [first quartile, third quartile], *n* = 2792 landings), and for landings following free flight, this was *U*_start_ = 0.34 [0.21, 0.49] m/s (*n* = 7213 landings) (Figure S2). The free flight landings were thus initiated at flight speeds similar to those observed in previous bumblebee studies and sometimes even surpassed them (Reber et al., 2015; Chang et al., 2016).

Video S1. Example of a bumblebee taking off from one platform and landing on another This movie is corresponding to photomontage in Figure 2D and plays 5.83x slower than the actual speed.

### The average flight kinematics of all recorded landing maneuvers

For each landing approach, we calculated the temporal dynamics of the following state variables (Figures 2D–2F): 3D position (*x*(*t*), *y*(*t*), *z*(*t*)), approach velocity (*V*(*t*) = –d*y*(*t*)/d*t*) and approach acceleration (*A*(*t*) = –d^2^*y*(*t*)/d*t*^2^) perpendicular to the landing platform, and the relative rate of expansion that a bumblebee experiences due to its motion perpendicular to the landing platform (*r*(*t*) = *V*(*t*)/*y*(*t*)).

On average, bumblebees performed the landing maneuver in a direction perpendicular to the platform (Figures 3A and 3B). During their mean landing maneuver, they advanced toward the platform by first gradually increasing their approach velocity (*V*), followed by a deceleration phase during which they decreased their approach velocity (0.04 m ≤ *y* ≤ 0.11 m). As previously observed in honeybees (Baird et al., 2013) and suggested for bumblebees (*B. impatiens*) (Chang et al., 2016), the average decelerating bumblebee decreased its approach velocity approximately linearly with distance, thus keeping the relative rate of expansion nearly constant at a set point *r*^∗^ (Figure 3C).

We used a linear mixed-effects model to test how this set point of the relative rate of expansion *r*^∗^ differed between tested treatments (light condition and landing platform) and between landings following takeoff and free flight (landing type) (see methods). This showed that the set point of the relative rate of expansion *r*^∗^ differed significantly between both light conditions and landing type, but *r*^∗^ did not differ between the landing patterns (Table S2). The relative rate of expansion set point was higher in brighter light conditions (Figures 3C, 3D, and S3), and it was higher in landings after takeoff than in landings from free flight (Figure S3). It implies that, in the presence of brighter light conditions and when the landing followed takeoff, bumblebees decelerated more quickly during the landing maneuver, thus allowing for higher approach velocities and more rapid landings.

The expansion rate set points of the landing maneuvers across all tested conditions were on average *r*^∗^ = 2.32 [0.24] s^−1^ and *r*^∗^ = 3.02 [0.24] s^−1^ for the free flight landings and the landings following takeoff, respectively (mean [standard error], *n* = 6 conditions). These values are similar to the expansion rate set points observed in landings of honeybees (Baird et al., 2013) and the set points suggested for *Bombus impatiens* landings (Chang et al., 2016).

### The flight kinematics of individual landing maneuvers

**Figure 4:**
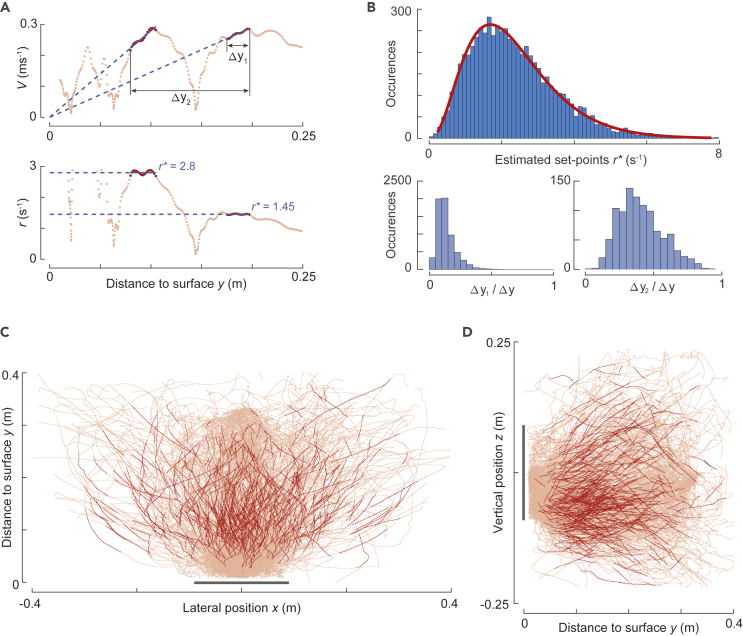
Landing bumblebees decelerate at a range of set points of relative rate of optical expansion (*r*^∗^). (A) The variation of approach velocity *V* and relative rate of expansion *r* with perpendicular distance from the platform *y* of the landing maneuver in Figures 2D–2F. The segments in which *r* is identified as (nearly) constant are highlighted in dark red. The corresponding relative rate of expansion set points *r*^∗^ are indicated by the dashed blue lines (as slope and ordinate values in the *V*-*y* and *r*-*y* graphs, respectively). (B) Top panel: histogram of the set points of relative rate of expansion *r*^∗^ for all identified constant-*r* segments (*n* = 6291 segments). Bottom panels: histograms of the ratio of displacement traveled by bumblebees during constant-*r* segments (Δ*y*_1_ or Δ*y*_2_) to the total displacement normal to the landing platform (Δ*y*). Left shows the relative distance traveled during a single constant-*r* segment Δ*y*_1_/Δ*y* (*n* = 6291 segments), and right shows the relative distance traveled during two consecutive constant-*r* segments Δ*y*_2_/Δ*y* (*n* = 1015 segments), as defined in the panel (A). (C and D) Top and side views of 470 tracks (every 10^th^ of 4672 tracks) used to study the landing dynamics. The complete flight tracks are shown in orange, and the track segments in which the optical expansion rate is kept constant are highlighted in red. The landing platform is shown in gray. (See also Figures S1 and S4 and Table S3).

Although the average approach dynamics suggests that bumblebees use a constant relative rate of expansion landing strategy as described previously (Chang et al., 2016), we observed that individual flight trajectories deviated often significantly from the average constant relative rate of expansion track (Figure 3C). In fact, many landing maneuvers consisted of multiple deceleration phases (Figures 2D–2F) instead of a single continuous one. To analyze these individual flight maneuvers separately, we used an in-house developed automatic detection algorithm to extract the segments of the landing maneuvers in which bumblebees kept the relative rate of expansion constant (Figure 4A, see methods for details).

Hereafter, we refer to the track segments identified using our detection algorithm as *constant-r segments* and characterize them by their average values of the four state variables (*y*^∗^*,V*^∗^*,A*^∗^*,r*^∗^), displacement normal to the platform (along *y* axis) during a single constant*-r* segment (Δ*y*_1_) and displacement normal to the platform for a set of consecutive constant-*r* segments (Δ*y*_2_) (Δ*y*_1_ and Δ*y*_2_ are annotated in Figure 4A). We use *r*^∗^ as an estimate of the set point of the relative rate of expansion that the bumblebee aims to hold constant (see methods for explanation).

The output of the constant*-r* detection algorithm depends on a setting parameter *f*, whereby higher *f* leads to the detection of more (and wider) constant-*r* segments and thus fewer false negatives and more false positives (see methods for details). We therefore performed a sensitivity analysis by systematically varying the factor *f* from 0.25 to 2.5 to determine its effect on the distribution of set points identified and their dynamics with distance described later in this section (see methods).

For *f* = 1, we identified 6,291 constant-*r* segments within the 4,672 landing maneuvers (1359 and 3313 landings starting from takeoff and free flight, respectively) out of a total of 10,005 maneuvers (Figures 4B–4D and S1). For *f* = 2.5, the number of constant-*r* segments increased to 16,322 constant-*r* segments identified within 7951 landing maneuvers. Although the number of constant-*r* segments increased with *f*, the distribution of constant-*r* segments (including their dynamics with distance) remained essentially unaltered throughout our tested range of *f*, so here, we report all results for factor *f* = 1 (see methods and Table S1 for results at the other *f* values).

### Landing maneuvers consist of multiple flight segments with constant-*r*

The set points of the relative rate of expansion varied considerably among segments (Figures 4B and S4), and their observed distribution can be approximated by the gamma distribution (median *r*^∗^ = 2.15 s^−1^, *a* = 3.59 [3.47–3.71], *b* = 0.65 [0.63–0.67], mean [95% confidence intervals], see methods for details).

For the 6,291 identified constant-*r* segments, the displacement during a single segment (Figure 4B) was Δ*y*_1_ = 0.035 ± 0.017 m (mean ± standard deviation), which consisted on average of only 13% of the total displacement (along *y*-direction) during the complete approach maneuver (Δ*y* = 0.266 ± 0.063 m, Figure 4B). This suggests that bumblebees, while approaching a landing platform, do not fly at a single set point of the relative rate of expansion, like observed in the average-per-treatment analysis. Instead, they fly at a constant relative rate of expansion for relatively short travel distances (0.035 m), after which they likely switch to a new set point of the relative rate of expansion.

### Landing bumblebees increase the constant-*r* set points when approaching the landing platform

**Figure 5:**
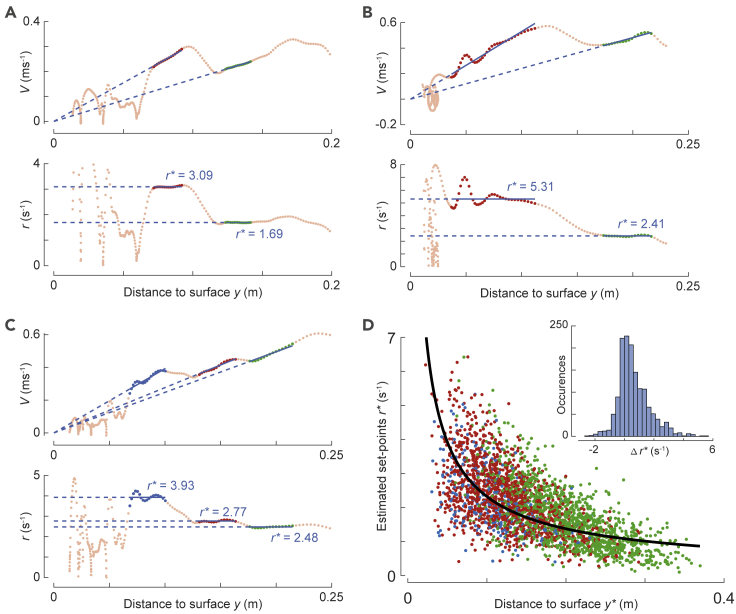
Bumblebees stepwise increase their set points of relative expansion rate during a landing approach. (A–C) Examples of landing approaches that start from a free-flight condition and contain multiple set points of relative rate of expansion, as shown by the variation of approach velocity *V* and relative rate of expansion *r* with perpendicular distance from the platform *y*. The track segments in which *r* is approximately constant are highlighted in green (first observed set point), red (second set point), and blue (third or higher set point). The magnitudes of the relative rate of expansion set points *r*^∗^ are indicated by the dashed blue lines (as slope and ordinate values in the *V*-*y* and *r*-*y* graphs, respectively). (D) Variation of relative rate of expansion set point *r*^∗^ with distance from the platform *y*^∗^ for landing maneuvers with multiple constant-*r* segments. The first set point in each track is shown in green, the second set point in red, and third or higher set points in blue. The average variation of *r*^∗^ with *y*^∗^ as estimated from the linear mixed-effects model is shown in black (see methods). The inset shows a histogram of the change in relative rate of expansion set point between two consecutive constant-*r* segments Δ*r*^∗^ (*n* = 1456). (See also Tables S3 and S4).

We tested how bumblebees adjusted these set points of the relative rate of expansion within a landing approach by analyzing the transitions from one set point to the next, for all landing maneuvers in which we detected multiple constant-*r* segments (Figure 5). Out of 4,672 landing maneuvers, 1015 maneuvers were identified with two constant-*r* segments (examples in Figures 5A and 5B) and 283 maneuvers with three or more constant-*r* segments (example in Figure 5C).

The displacement during two consecutive constant-*r* segments (Figure 4B) was on average Δ*y*_2_ = 0.114 ± 0.049 m (*n* = 1015 landings) and thus explained on average 40% of the total approach displacement (Figure 3D). Because the mean approach displacement during a single constant-*r* segment (Δ*y*_1_) was 13%, bumblebees traversed approximately 1/3^rd^ of the displacement during two consecutive constant-*r* segments (Δ*y*_2_) while transitioning from one set point to another. Of all transitions between two consecutive constant-*r* segments, 72% of them were from a lower constant-*r* set point value to a higher value, and the average set point increase was Δ*r*^∗^ = 1.05 ± 0.93 s^−1^ (*n* = 1050 transitions). Thus, during a transition, bumblebees tend to increase the set point of the relative rate of expansion (on average 113%), and a set of two consecutive constant-*r* segments represents a significant proportion (40%) of the total displacement during a landing maneuver. These results are consistently observed for each tested treatment and for both landing types (after takeoff and from free flight) (Table S3).

### The stepwise increase of the relative rate of expansion set points occurs at a constant time-to-contact rate

**Figure 6:**
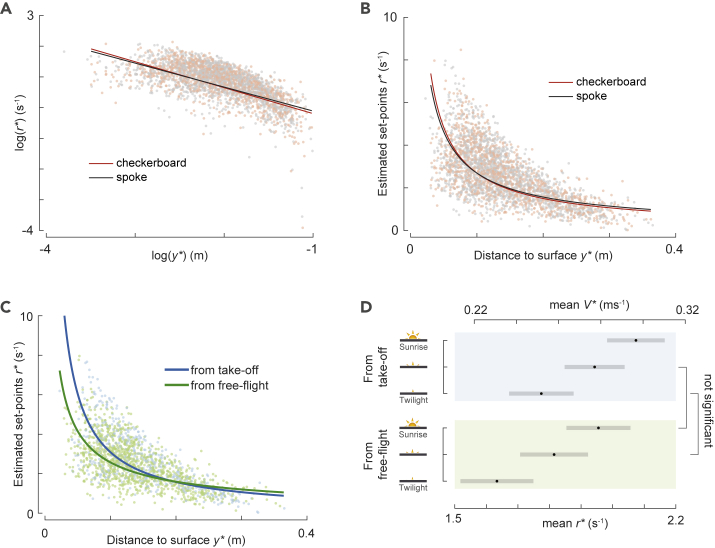
In all tested conditions, landing bumblebees stepwise increase their set points of relative expansion rate such that they approximate a constant time-to-contact rate landing strategy. (A and B) The set points of relative rate of expansion *r*^∗^ versus perpendicular distance from the landing platform *y*^∗^, for all detected constant-*r* segments in landings of freely-flying bumblebees in sunrise light, when landing on a checkerboard platform (red) or a spoke platform (gray). Data are shown in the log-transformed domain (A) and the untransformed domain (B). (C) Variation of *r*^∗^ with *y*^∗^ for all detected constant-*r* segments of landings on a spoke landing platform in sunrise light, when initiated from takeoff (blue) and from free flight (green). (A-C) Data points show all detected constant-*r* segments in the condition defined by color, and the solid lines show the linear mixed-effects model fits in the log-transformed domain for the same condition. (D) The relative rate of expansion *r*^∗^ and approach flight speed *V*^∗^ at the average distance from the landing platform (*y*^∗^ = 0.15 m), as predicted by the model for landings directly after takeoff (top) and from free flight (bottom), and at low, medium, and high light conditions. The approach speed at *y*^∗^ = 0.15 m increases with increasing light intensity and is higher for landings initiated after takeoff (Table S5 for *p* values). Black dots depict estimated means and gray bars are 95% confidence intervals. Non-significant differences are indicated on the right. Equivalent data for the other combinations of environmental conditions and landing types are available in Table S5.

The dynamics of increasing set points of the relative rate of expansion with decreasing distance from the landing platform (Figure 5) resembles the trend observed in birds that use the constant $\dot{\text{τ}}$ landing strategy (Figure 1C). We tested whether bumblebees comply to this strategy by fitting a linear mixed-effects model (see methods) to both the data set with the maneuvers containing multiple constant-*r* segments (Figure 5D, Table S4, n = 2917 segments) and the complete data set (Figures 6A–6C, Table S5, n = 6,291 segments). The model predicts an average time-to-contact rate $\dot{\text{τ}}$ = −0.78 and $\dot{\text{τ}}$ = −0.87 for the reduced data set and complete data set, respectively. Thus, bumblebees increase the set points of the relative rate of expansion while approaching the landing platform at a constant time-to-contact rate. The resulting average time-to-contact rate at which they do this is strikingly similar to that observed in birds ($\dot{\text{τ}}$ = −0.76 for hummingbirds [Lee et al., 1991] and $\dot{\text{τ}}$ = −0.72 for pigeons [Lee et al., 1993]).

Our linear mixed-effects model analysis also allowed us to test how the landing strategy differed with light intensity, optical expansion information of the landing platform, and between landings performed from free flight and landings that followed takeoff (Figure 6, Table S5). The minimal linear mixed-effects model included effects of all treatments and landing type but no interactions between these (see methods for details). Therefore, we here discuss the effects of light intensity, landing pattern, and landing type consecutively.

### In high light intensities, bumblebees approach the landing platform at higher speeds

Our linear mixed-effects model analysis shows that the relative rate of expansion set point differed significantly between all light conditions, but the set point *r*^∗^ did not differ significantly with the interaction between light intensity and *y*^∗^ (Table S5). As a result, the model predicts that for an average landing maneuver, the relative rate of expansion set point at the average distance *y*^∗^ = 0.15 m equals *r*^∗^ = 1.68 [0.05] s^−1^, *r*^∗^ = 1.82 [0.05] s^−1^, and *r*^∗^ = 1.99 [0.05] s^−1^ in low, medium, and high light condition, respectively (mean [standard error]). This corresponds to an average approach flight speed of *V*^∗^ = 0.25 [0.01] m s^−1^, *V*^∗^ = 0.27 [0.01] m s^−1^, and *V*^∗^ = 0.30 [0.01] m s^−1^ in low, medium, and high light condition, respectively. In contrast, the variation of *r*^∗^ with *y*^∗^ did not change with light condition, showing that the governing time-to-contact rate for adjusting set points with distance did not change with light intensity. Thus, bumblebees approached the landing platform on average 19% faster in the bright sunrise light conditions than in twilight, but the bumblebees slowed down at a similar rate in various light conditions.

### Bumblebees brake more rapidly when landing on a platform with low optical expansion information

The statistical results related to the effect of landing platform pattern on the landing dynamics is opposite to that of light intensity: the relative rate of expansion set point at the mean distance *y*^∗^ = 0.15 m did not differ significantly between landings on the different platforms but did differ significantly with the interaction between landing pattern and distance from the platform *y*^∗^ (*p* = 0.00025, Table S5). Based on this, the statistical model predicts that, for an average bumblebee, the time-to-contact rate is 11% smaller when approaching a checkerboard landing platform with high optical expansion information ($\dot{\text{τ}}$ = −0.92 [0.02]) than when approaching a spoke landing platform with low optical expansion information ($\dot{\text{τ}}$ = −0.83 [0.02]).

This shows that bumblebees approaching a spoke landing platform with low optical expansion cues slowed down more quickly (higher $\dot{\text{τ}}$) than bumblebees landing on a checkerboard platform with high optical expansion information. This results in lower approach speeds close to the landing platform, suggesting that bumblebees land more carefully on the less conspicuous platform.

### Bumblebees landing from free flight brake more rapidly than bumblebees landing after takeoff

We finally tested how the landing strategy differed between landings that were performed directly after taking off and landings from free flight. The linear mixed-effects model shows that the relative rate of expansion set point differed significantly with both landing type and the interaction between landing type and *y*^∗^ (*p* < 0.0001, Table S5). The model predicts that an average bumblebee slowed down more quickly (higher $\dot{\text{τ}}$) when landing from free flight ($\dot{\text{τ}}$ = −0.73 [0.01]) than when landing after takeoff ($\dot{\text{τ}}$ = −1.01 [0.02]) (Figure 6C). This shows that bumblebees that land from free flight start their approach flight at a higher approach velocity, but because they decelerate more quickly, they end their approach at a lower approach velocity (Figure 6C). As a result of this rapid deceleration, the approach velocity close to the platform (*y*^∗^ = 0.05 m) is 31% lower when landing from a free flight (*V*^∗^ = 0.197 [0.006] m s^−1^) than when landing after takeoff (*V*^∗^ = 0.287 [0.010] m s^−1^).

## Discussion

Here, we studied how bumblebees (*B. terrestris*) decelerate to land smoothly when performing foraging flights. This includes landings that directly follow after taking off and landings that are initiated from a free flight. These two landing types represent two common but possibly distinct landing maneuvers. The landing directly after takeoff is performed by foraging bumblebees at rates of up to a 1000 times per hour when moving between flowers in a single flower patch (Heinrich, 1979); the landing from free flight is commonly performed when moving between flower patches and the hive.

To study the landing dynamics of both types of maneuvers, we trained bumblebees to forage for food. They landed on two vertical platforms directly from free flight (7213 landings) or after taking off from the opposite platform or ground (2792 landings). We placed the landing platforms 0.34 m apart, similar to the average 0.33 m distance between consecutively visited fresh flowers by bumblebees within a patch (Heinrich, 1979).

We systematically varied the visual pattern on the landing platforms (low and high optic expansion information), and we varied the environmental light intensities from twilight to sunrise conditions. Although we use vertical landing platforms, the deceleration strategy described in our study is based on an optic flow profile generated for landings on surfaces of any orientation and for any direction of approach (Baird et al., 2013).

### Average landing approach kinematics versus approach kinematics of individual bumblebees

To examine how bumblebees decelerated during a landing approach, we use two different analysis methods, referred to as average-per-treatment and per-track analysis methods.

In the average-per-treatment method, we first analyzed the mean of all 10,005 approaches and selected a range of distance interval (0.04 m ≤ *y* ≤ 0.11 m) in which the approach velocity toward the platform decreased proportionately with distance. Within this distance range, we then analyzed how the mean relative rate of expansion varied with tested treatment (landing pattern and light intensity) and between landing type (from takeoff or from free flight).

In the per-track analysis method, we first extracted the segments in which a bumblebee kept its relative rate of expansion constant (this constant is referred to as the relative rate of expansion set point) within each landing approach and then analyzed how the mean relative rate of expansion of all identified segments varies with the distance to the landing surface, for different treatments, and between landing types. It should be noted that the per-track analysis is inclusive of average-per-treatment analysis, i.e., if individual landing maneuvers are similar to the average landing approach, per-track analysis will identify constant-*r* segments only near the distance interval selected for the average-per-treatment analysis and will yield negligible dependence of set point on distance.

Using the average-per-treatment analysis – a method used in previous studies (Baird et al., 2013; Chang et al., 2016) – we found that bumblebees on average approached the platform by first increasing their velocity and then decelerated by decreasing their velocity linearly with distance to make a soft touchdown. This suggests that our bumblebees approach the landing platform by flying at a constant relative rate of expansion, as has also been described in honeybees (Baird et al., 2013) and suggested in other bumblebees (Chang et al., 2016).

The mean set point of relative rate of expansion differed between light conditions and landing type, but not between landing platforms (Figures 3C, 3D, and S3). At higher light intensities and for landings initiated from takeoff, the rate of expansion set point was higher, resulting in a higher mean approach velocity. As a result, landings after takeoff were on average 30% faster than landings initiated from free flight, and landings in the highest light condition (sunrise) were on average 29% faster than in the lowest twilight condition.

The mean expansion rate set point at sunrise is similar to that of honeybees (8% lower for the checkerboard pattern and 15% lower for the spoke pattern) (Baird et al., 2013), but 34% lower than for *B. impatiens* bumblebees (unreported and estimated from Figure 2A in Chang et al., 2016). This striking difference in expansion rate set point could be due to differences in light conditions, as we here showed that light intensity affects the relative rate of expansion set point. However, we cannot test this because light intensity was not reported in the previous studies (Baird et al., 2013; Chang et al., 2016). A second explanation could be the differences in maximum distance available in front of the landing platform, which was 0.41 m in our setup, 1.5 m for the honeybees study (Baird et al., 2013), and 6 m for the *B. impatiens* study (Chang et al., 2016). Although our free-flight landings are initiated at speeds similar to those of *B. impatiens* (Chang et al., 2016), a new study in which landing distance is varied systematically would be needed to test this.

### The hybrid landing strategy of short-distance landing maneuvers in bumblebees

The average-per-treatment analysis provided a useful insight into the mean approach dynamics but failed to capture the approach dynamics of all individual landing maneuvers. Specifically, it missed the deceleration phases that were spread across a landing approach. To capture all deceleration phases, we used our custom-developed per-track analysis method. Using this analysis, we extracted 6,291 segments (within the 4,672 landings) in which individual bumblebees kept their relative rate of expansion constant (constant-*r* segments); for each segment, we estimated the relative rate of expansion set point at which the animal flew. The distribution of these 6,291 set points reveals that landing bumblebees exhibit a skewed distribution of set points in all tested treatments (Figures 3C and S5). The observed distribution of set points encompasses the set points from average-per-treatment analyses and the ones observed for honeybees and bumblebees in earlier studies (Baird et al., 2013; Chang et al., 2016).

To determine the switching dynamics of constant-*r* set points within a landing approach, we analyzed the set point variation with distance for 1,298 approaches in which we detected more than one constant-*r* segment (Figure 5D). We found that, within a landing approach, bumblebees most often switched from a lower set point of relative rate of expansion to a higher one as this was the case in 72% of all observed transitions, and the average set point after transition was 113% higher than before. This shows that the observed wide range of set points of relative rate of expansion is not due to the individual differences between bumblebees but that the bumblebees can exhibit more than one constant-*r* set point within a single landing approach. Moreover, these dynamics are very similar between tested treatments and landing types (Table S3), indicating that the internal process of switching the set points within a landing approach happens with the same probability irrespective of both environmental conditions and landing type.

To determine how bumblebees collectively adjusted their relative rate of expansion set points as they approached the landing platforms, we tested the variation of set points of relative rate of expansion (*r*^∗^) with distance to the platform (*y*^∗^) for the 6,291 detected constant-*r* segments. We found a linear relationship between the log transformations of *r*^∗^ and *y*^∗^, suggesting that bumblebees increase their set points during deceleration at a constant time-to-contact rate (Figures 1B, 1C, and 6A). These estimates of time-to-contact rate in bumblebees varied from −0.690 to −1.054 for all tested treatments and landing types and are thus similar to those observed in hummingbirds ($\dot{\text{τ}}$ = −0.76) (Lee et al., 1991) and pigeons ($\dot{\text{τ}}$ = −0.72) (Lee et al., 1993) (the reported time-to-contact rates from literature are transformed to sign convention depicted in Figure 2C). The key difference between deceleration strategies of bumblebees and birds is that birds regulate their relative rate of expansion continuously at a negative time-to-contact rate, whereas bumblebees adjust the set points of relative rate of expansion in steps at a negative time-to-contact rate, thereby discretely approximating the constant-$\dot{\text{τ}}$ strategy of birds (Figure 1).

The adjustment of set points with distance is observed in data sets with landing maneuvers in which we detected only multiple constant-*r* segments (Table S4), only a single constant-*r* segment (Table S6) and when both data sets were pooled together (Table S5). This strongly indicates that tracks containing only one constant-*r* segment may also have more constant-*r* segments that were not detected due to the limitations of our constant-*r* extraction method. These limitations can occur due to the factor *f* that restricts the variation of *r* allowed in a constant-*r* segment (see methods), or the fundamental basis of the per-track analysis methodology that detects only the set points that bumblebees have been able to reach and follow in their trajectory. We overcome both of these limitations by using a large data set with thousands of landing approaches.

Here, we conclude that bumblebees effectively use a hybrid between the constant-*r* landing strategy described in honeybees (Baird et al., 2013) and the constant-$\dot{\text{τ}}$ landing strategy observed in birds (Lee et al., 1991; Lee et al., 1993), as they exhibit several segments of constant-*r* and regulate the set points of these constant-*r* segments in a constant-$\dot{\text{τ}}$ manner.

### The hybrid landing strategy is faster than a constant-*r* landing strategy

It has been suggested that the constant-$\dot{\text{τ}}$ deceleration strategy used by birds results in faster approach flights than the constant-*r* strategy used by honeybees (Baird et al., 2013). We tested how the here-described hybrid landing strategy compares to both strategies. For this, we calculated for the 1008 landings with two consecutive constant-*r* segments, the hybrid-to-constant-*r* speed ratio and the hybrid-to-constant-$\dot{\text{τ}}$ speed ratio as $U_{\text{H}}/U_{\text{r}}$ and $U_{\text{H}}/U_{\dot{\text{τ}}}$. Here, $U_{\text{H}}$ is the average flight speed during the combined flight segment, and $U_{\text{r}}$ and $U_{\dot{\text{τ}}}$ are the equivalent speeds if the bumblebee would have used the constant-*r* and constant-$\dot{\text{τ}}$ strategy, respectively (see methods for details). The constant-*r* and constant-$\dot{\text{τ}}$ are based on the first set point in a set of two consecutive constant-*r* segments and average time-to-contact rate observed in our data set, respectively. For 1008 landings with two detected constant-*r* segments, the hybrid-to-constant-*r* speed ratio is $U_{\text{H}}/U_{\text{r}}$ = 1.16 ± 0.69 and the hybrid-to-constant-$\dot{\text{τ}}$ speed ratio is $U_{\text{H}}/U_{\dot{\text{τ}}}$ = 0.88 ± 0.55. This shows that the here-described hybrid landing strategy of bumblebees is 16% faster than if the bumblebee would use the equivalent constant-*r* strategy, but 12% slower than if it would continuously fly at a constant-$\dot{\text{τ}}$. The reduction in effective flight speed relative to the true constant-$\dot{\text{τ}}$ observed in birds is because bumblebees keep relative rate of expansion constant for some time and experience transition dynamics between two consecutive set points.

### Robustness of the hybrid landing strategy of bumblebees

To test the robustness of the hybrid strategy, we offered the bumblebees different light conditions ranging from twilight to sunrise, and allowed them to land on two different landing platforms, one with a checkerboard pattern and one with a spoke pattern. We find that bumblebees robustly exhibit this strategy in all tested conditions, but with significant differences.

During constant-*r* segments, our statistical model predicts that at the average distance from the landing platforms (*y*^∗^ = 0.15 m), bumblebees fly slower at lower light conditions, with differences ranging from 8% to 18% among different tested light conditions and for two landing types (Figures 6D, Table S5). However, the slope ($\dot{\text{τ}}$ estimates) of regulating the set points (*r*^∗^) with distance (*y*^∗^) is not significantly different between light conditions. This shows that bumblebees tend to fly at lower speeds under lower light intensity but that the governing set point dynamics does not change with light condition. This finding is similar to the results from our average-per-treatment analysis (Figure 3D) and suggests that as light intensity falls, bumblebees possibly use neural temporal summation to improve the reliability of visual cues, and they thus fly slower to compensate for the resulting loss of temporal resolution (Baird et al., 2015; Reber et al., 2015). It is congruent with the observation in cruising flights of bumblebees (*B. terrestris)* (Reber et al., 2015) where they also reduce their mean flying speed with a decrease in light intensity.

In contrast to the negligible effect of light on set-point dynamics, the visual expansion information of the landing platform does affect the effective time-to-contact rate at which the landing bumblebees change their set points. When approaching the spoke landing platform with low visual expansion information, the bumblebees fly at 10% higher time-to-contact rate than when approaching the checkerboard platform with high expansion information. As a result, bumblebees approaching a landing platform with limited optic expansion cues decelerate more rapidly, which results in a lower approach velocity when they reach the landing platform, thus reducing the chance of collision with the surface. Because theoretically *r*^∗^ can be set in the brain of a bumblebee, independent of *r* (a sensory measurement), bumblebees slow down more quickly and, thus, perform a more careful landing when less visual expansion information is present. These results are similar to the behavior described in honeybees where they approached a spoke landing platform at a 4% lower average relative rate of expansion than a checkerboard pattern (Baird et al., 2013).

### Differences in the landing strategy between landings from free flight and after takeoff

In our study, we recorded two types of landing maneuvers, the landing directly after a takeoff and the landing initiated from free flight. We tested how these two landing types that are both commonly performed by foraging bumblebees differ.

We find that, for both types of landing maneuvers, bumblebees use the here-described hybrid landing strategy, but especially the time-to-contact rates that govern set point adjustment with distance are strikingly different (Figure 6C). Bumblebees that land from free flight exhibited on average a 28% higher time-to-contact rate than when they landed directly after takeoff. Moreover, bumblebees that land from free flight start their landing maneuver at a higher approach velocity, but because they decelerate more quickly (with 28% higher time-to-contact rate), their approach velocity at landing is much lower than for the landings after takeoff (31% lower speed at *y*^∗^ = 0.05 m). This shows that landings from free flight are performed much more carefully than landings following takeoff, similarly to landings on a platform with low and high visual expansion cues, respectively.

The fact that these rapid consecutive takeoff and landing maneuvers are performed much more commonly by the foraging bumblebees could explain these differences as bumblebees might have learned to perform such frequent landings both rapidly and safely. A similar type of learning has been described in foraging honeybees, where honeybees that forage in an unfamiliar environment improve their in-flight aerodynamic braking in time to increase their landing success (Muijres et al., 2020).

### Differences in the landing strategy between honeybees and bumblebees

Considered together, our results describe a deceleration strategy of *Bombus terrestris* during landing that is different from the deceleration strategy suggested previously for *Bombus impatiens* (Chang et al., 2016) and observed in *Apis mellifera* (Baird et al., 2013). These differences could exist due to differences among species, tested light conditions, maximum distance available in front of the landing platform, or analysis methods. It is unlikely that the differences in distance available in front of the platform is the primary cause because the bumblebees in our setup flew at approach velocities similar to the cruising speeds reported in previous bumblebee studies (Reber et al., 2015; Chang et al., 2016) (Figures 5A–5C). Light condition could possibly explain differences in the magnitude of the relative rate of expansion set point, but it is unlikely that it explains the difference between the hybrid and constant-*r* landing strategy among studies. Thus, the differences among species and analysis method are the most likely candidates for explaining the occurrence of two distinct landing strategies. Because our per-track analysis is more comprehensive than the average-per-treatment analysis used in literature, it would be interesting to apply our analysis method to the landing dynamics of *Apis mellifera ligustica* and *Bombus impatiens* to rule out any effect of analysis methods on observed deceleration strategies.

There is one previous honeybee landing study that used an individual track-based analysis method (Srinivasan et al., 2000). This study showed that when honeybees land on a horizontal surface, they reduce their forward flight speed linearly with distance to the surface and, thus, do not make use of the here-described hybrid landing strategy. This suggests that the landing strategy difference between our study and that described in literature (Srinivasan et al., 2000; Baird et al., 2013; Chang et al., 2016) is due to differences in species. But note that forward-flying honeybees land on horizontal surfaces by regulating front-to-back translatory optic flow, instead of optic expansion cues (Baird et al., 2013). Therefore, to conclusively determine the cause of the differences in landing strategies used by our bumblebees and honeybees, one would need to apply our analysis method to the landings that honeybees control using optical expansion cues.

### How do bumblebees execute the hybrid landing strategy?

There is another important remaining question: during the hybrid landing approach, what triggers switching from one constant-*r* set point to another? This question is especially relevant because optical flow cues, such as visual expansion, capture the ratio of velocity and distance but do not allow disentangling these quantities. The dynamics of the transitions may provide a clue here. Most transitions look relatively smooth, but especially when closer to the landing surface, oscillations in *r* around the set point are evident (Figure 5). Moreover, among the 1015 tracks containing two constant-*r* segments, as many as 23% of the transitions contained near-zero approach velocity (*V* < 0.05 m s^−1^). These observations point to the direction of a recent theory on monocular distance perception (de Croon, 2016), which postulates that insects can detect the instabilities that arise when performing closed-loop optical flow control. It was shown that (de Croon, 2016), given a fixed control system, these instabilities arise at specific distances from the target object, allowing disentangling distance and speed. In the current case, the detection of instabilities could provide the bumblebee an estimate of distance to the surface which consequently could trigger the change in set point. However, alternative explanations are possible, such as the use of other distance cues (de Croon et al., 2021) or parallax cues arising from lateral motion (Baird et al., 2021). More research is needed to shed further light on this essential part of the hybrid landing strategy.

### How can bumblebees estimate the relative rate of expansion?

The studies depicting neural measurements of relative rate of expansion (or time to contact) are scarce and we are aware of only one example of computation of threshold time-to-contact value by the neural system in pigeons (Wang and Frost, 1992; Sun and Frost, 1998). However, when an animal approaches a surface, it can use some measure of absolute-rate of expansion (or simply, rate of expansion ρ) (e.g., ρ averaged over a part of visual field, maximum ρ in the visual field) as a proxy for relative rate of expansion (Baird et al., 2013). Also, neural measurements of absolute rate of expansion have been recorded in honeybees (Ibbotson et al., 2017). It is therefore likely that certain neurons in bumblebees' visual neuropil also measure absolute rate of expansion which could then be used as an alternate for relative rate of expansion.

### Conclusion

By using our custom-designed individual-track-based analysis method, we here described the deceleration strategy that bumblebees exhibit during landing. Specifically, we have shown that landing bumblebees decrease their velocity toward the landing platforms by holding the relative rate of optic expansion cue constant for only short bouts within the landing maneuver. From one bout to the next, they tend to increase the optic-expansion set point at which they fly. This modular increase in set points with reducing distance results in a discrete approximation of the deceleration strategy of birds. Birds use a constant time-to-contact rate to regulate their expansion rate with distance, which results in relatively fast landings.

The landing strategy of bumblebees is observed in the presence of variable degrees of optic expansion cues and is exhibited by bumblebees landing both after takeoff and from free flight. Moreover, it occurs in a wide range of luminance levels, suggesting that bumblebees adequately control landing by using neural summation. Our results are a step toward detailed understanding of how bumblebees robustly control their landing approaches. Once sufficiently understood, these control strategies can provide bioinspiration for the development of landing algorithms in autonomously flying robots.

### Limitations of the study

#### Limitations of our relative rate of expansion set point analysis method

Our analyses assume that the sensorimotor control system of landing bumblebees sets the set points of relative rate of expansion as a goal in the brain of the bumblebee. This assumption needs to be further investigated and supported by neuroethological studies that are aimed at identifying the neural circuits that underlie this sensorimotor control system. The response property of set point identified in this study, i.e., its modulation with distance, can be useful for this purpose.

Based on the above assumption, we analyzed the landing dynamics of foraging bumblebees using two analysis methods, namely the *average-per-treatment* method and the *per-track* analysis method. Both methods provide useful insights but also have their specific limitations as described below.

#### Limitations of our analysis method based on the average landing dynamics

The average-per-treatment analysis method allows us to estimate the average set points of relative rate of expansion in each treatment group. This provided a useful insight into the mean approach dynamics of bumblebees and allows testing of how this differs between conditions (treatments). In contrast, the methods ignore the detailed landing dynamics exhibited by individual bumblebees. Specifically, it does not capture the rapid deceleration phases that occur in between phases at which the animal would aim to fly at a constant relative rate of expansion. To analyze these detailed flight dynamics, we developed our analysis method based on the individual flight trajectories.

#### Limitations of our analysis method based on the individual flight trajectories

The per-track analysis method allowed us to identify the hybrid landing strategy described in this study, but the method has one primary limitation. Because the per-track analysis method identifies set points of relative rate of expansion in individual flight trajectories, a set point can only be identified if the bumblebee flies at this set point for a certain time period. Bumblebees can fail to reach a set point for several reasons. For example, the animal can land before reaching it, the animal could switch to a new set point before reaching the previous set point, or a landing can be aborted prior to it. In our study, we identified 6,291 set points of relative rate of expansion (for *f* = 1) within 10,005 landing maneuvers, suggesting that bumblebees regularly do not fly at their expansion-rate set point.

The ability to detect a set point of relative rate of expansion depends on the sensitivity of our per-track analysis method, set by the *f*-factor (see methods). The number of identified set points increases with *f* factor, but also the number of false-positive set points increases. Based on our sensitivity analysis, we showed that the main conclusions of our study are relatively insensitive to the *f*-factor value (see methods).

In conclusion, our per-track analysis method does not allow us to identify all set points of relative rate of expansion during a landing maneuver. But because our analysis is based on a large number of flights, this does not limit us in identifying and accurately describing the hybrid landing strategy of bumblebees.

### Resource availability

#### Lead contact

Florian T. Muijres, De Elst 1, 6708 WD, Wageningen, the Netherlands (+31 317 486 977, florian.muijres@wur.nl)

#### Materials availability

All materials related to this paper have been included in the paper.

#### Data and code availability

The data gathered during experiments are available in Mendeley Data: https://dx.doi.org/10.17632/rrbjyhkm8z.1 and the code used in the analysis is available in https://github.com/kaku289/nimble-bbee-analysis/tree/rref.

## Methods

All methods can be found in the accompanying transparent methods supplemental file.

## Supplemental information

Document S1 Transparent methods, Figures S1–S7, and Tables S1–S8

## References

[bib1] Baird E., Boeddeker N., Ibbotson M.R., Srinivasan M.V. (2013). A universal strategy for visually guided landing. Proc. Natl. Acad. Sci. U S A.

[bib2] Baird E., Fernandez D.C., Wcislo W.T., Warrant E.J. (2015). Flight control and landing precision in the nocturnal bee Megalopta is robust to large changes in light intensity. Front. Physiol..

[bib3] Baird E., Boeddeker N., Srinivasan M.V. (2021). The effect of optic flow cues on honeybee flight control in wind. Proc. R. Soc. B Biol. Sci..

[bib4] Balebail S., Raja S.K., Sane S.P. (2019). Landing maneuvers of houseflies on vertical and inverted surfaces. PLoS One.

[bib5] Van Breugel F., Dickinson M.H. (2012). The visual control of landing and obstacle avoidance in the fruit fly *Drosophila melanogaster*. J. Exp. Biol..

[bib6] Chang J.J., Crall J.D., Combes S.A. (2016). Wind alters landing dynamics in bumblebees. J. Exp. Biol..

[bib7] Corbet S.A., Fussell M., Ake R., Fraser A., Gunson C., Savage A., Smith K. (1993). Temperature and the pollinating activity of social bees. Ecol. Entomol..

[bib8] de Croon G.C.H.E. (2016). Monocular distance estimation with optical flow maneuvers and efference copies: a stability-based strategy. Bioinsp. Biomim..

[bib9] de Croon G.C.H.E., De Wagter C., Seidl T. (2021). Enhancing optical-flow-based control by learning visual appearance cues for flying robots. Nat. Mach. Intell..

[bib10] Edwards M., Ibbotson M.R. (2007). Relative sensitivities to large-field optic-flow patterns varying in direction and speed. Perception.

[bib11] Fontaine C., Dajoz I., Meriguet J., Loreau M. (2006). Functional diversity of plant-pollinator interaction webs enhances the persistence of plant communities. PLoS Biol..

[bib12] Foster D.J., Cartar R.V. (2011). What causes wing wear in foraging bumble bees?. J. Exp. Biol..

[bib13] Gibson J.J. (1955). The optical expansion-pattern in aerial locomotion. Am. J. Psychol..

[bib14] Heinrich B. (1979). Resource heterogeneity and patterns of movement in foraging bumblebees. Oecologia.

[bib15] Ibbotson M.R., Hung Y.S., Meffin H., Boeddeker N., Srinivasan M.V. (2017). Neural basis of forward flight control and landing in honeybees. Sci. Rep..

[bib16] Joar Hegland S., Totland Ø. (2008). Is the magnitude of pollen limitation in a plant community affected by pollinator visitation and plant species specialisation levels?. Oikos.

[bib17] Lee D.N. (1976). A theory of visual control of braking based on information about time to collision. Perception.

[bib18] Lee D.N., Davies M., Green P. (1993). Visual control of velocity of approach by pigeons when landing. J. Exp. Biol..

[bib19] Lee D.N., Bootsma R., Frost B., Land M., Regan D., Gray R. (2009). Lee’s 1976 paper. Perception.

[bib20] Lee D.N., Reddish P.E., Rand D.T. (1991). Aerial docking by hummingbirds. Naturwissenschaften.

[bib22] Reber T., Vähäkainu A., Baird E., Weckström M., Warrant E., Dacke M. (2015). Effect of light intensity on flight control and temporal properties of photoreceptors in bumblebees. J. Exp. Biol..

[bib21] Muijres F.T., van Dooremalen C., Lankheet M., Lugt H., de Vries L.J., Van Langevelde F. (2020). *Varroa destructor* infestation impairs the improvement of landing performance in foraging honeybees. R. Soc. Open Sci..

[bib23] Reber T., Dacke M., Warrant E., Baird E. (2016). Bumblebees perform well-controlled landings in dim light. Front. Behav. Neurosci..

[bib24] Srinivasan M.V., Zhang S.W., Chahl J.S., Barth E., Venkatesh S. (2000). How honeybees make grazing landings on flat surfaces. Biol. Cybern..

[bib25] Straw A.D., Branson K., Neumann T.R., Dickinson M.H. (2011). Multi-camera real-time three-dimensional tracking of multiple flying animals. J. R. Soc. Interf..

[bib26] Sun H., Frost B.J. (1998). Computation of different optical variables of looming objects in pigeon nucleus rotundus neurons. Nat. Neurosci..

[bib27] Velthuis H.W., van Doorn A. (2006). A century of advances in bumblebee domestication and the economic and environmental aspects of its commercialization for pollination. Apidologie.

[bib28] Wagner H. (1982). Flow-field variables trigger landing in flies. Nature.

[bib29] Wang Y., Frost B.J. (1992). Time to collision is signalled by neurons in the nucleus rotundus of pigeons. Nature.

