# Supplementary material for: Bumblebees land rapidly and robustly using a sophisticated modular flight control strategy
Source: iScience. 2021 Apr 24;24(5):102407. doi: 10.1016/j.isci.2021.102407 (PMC8099750; doi:10.1016/j.isci.2021.102407)
Supplement: Document S1 Transparent methods, Figures S1–S7, and Tables S1–S8 [file mmc1.pdf]

iScience, Volume *24*

Supplemental information

Bumblebees land rapidly and robustly using a sophisticated modular flight control strategy

Pulkit Goyal, Antoine Cribellier, Guido C.H.E. de Croon, Martin J. Lankheet, Johan L. van Leeuwen, Remco P.M. Pieters, and Florian T. Muijres

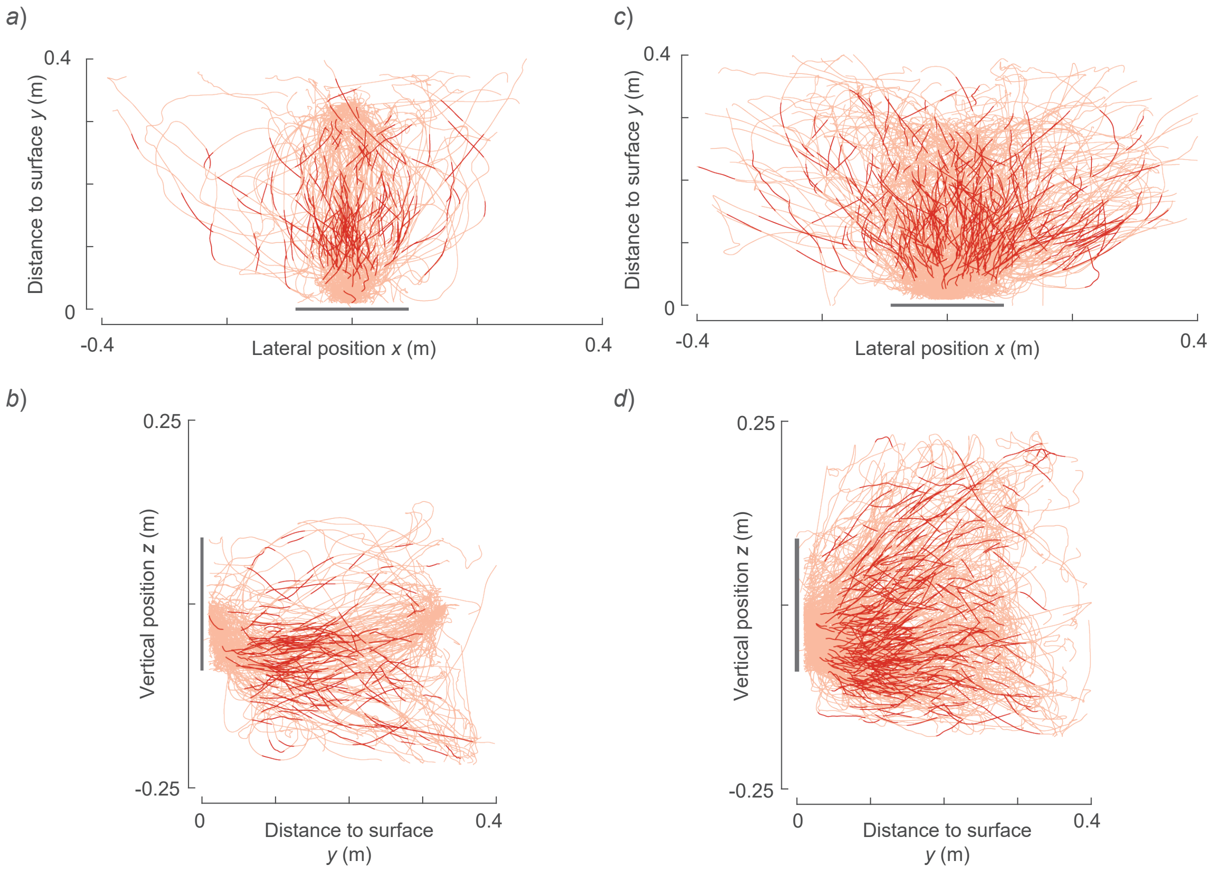

Figure S1: Flight trajectories of bumblebees landing directly after take-off (a,b) and from free-flight (c,d) (related to Figure 4). (a,b) Top and side views of 138 flight trajectories of bumblebees that landed immediately after taking-off from either the ground or the opposite platform (every 10th of 1359 flight tracks are shown). (c,d) Top and side views of 334 flight trajectories of bumblebees that initiated landing from a free-flight (every 10th track of 3313 recorded tracks are shown). (a-d) The flight tracks, landing platform, and the track segments in which optical expansion rate is kept constant are shown in orange, grey, and red, respectively.

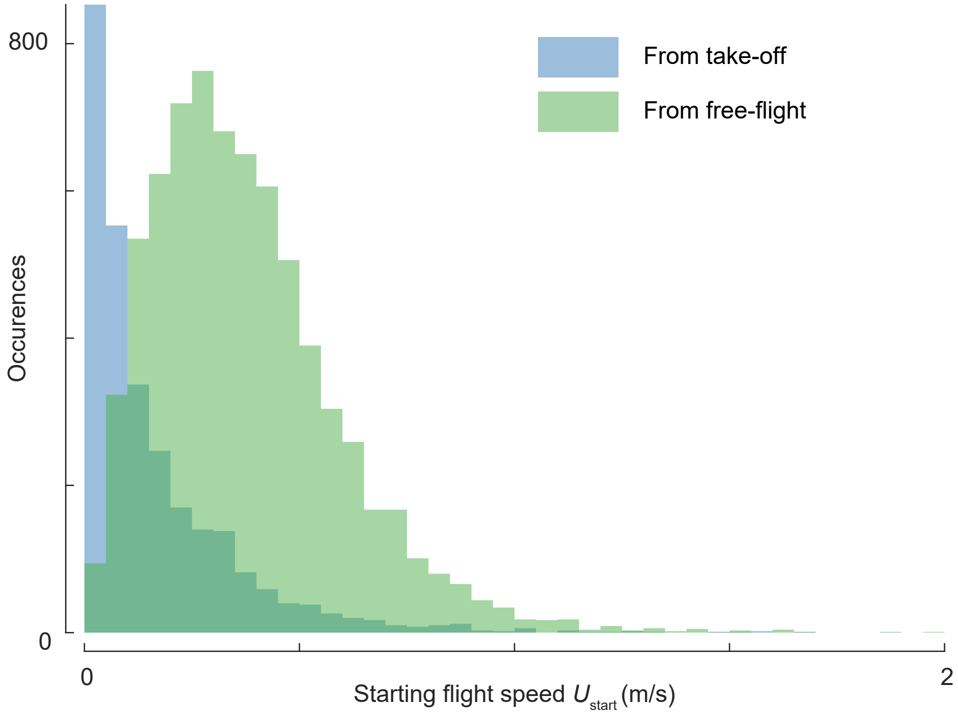

Figure S2: Histogram of flight speeds at the start of the landing manoeuvres performed by bumblebees from take-off (blue, n=2792) and from free-flight (green, n=7213) (related to Figure 3).

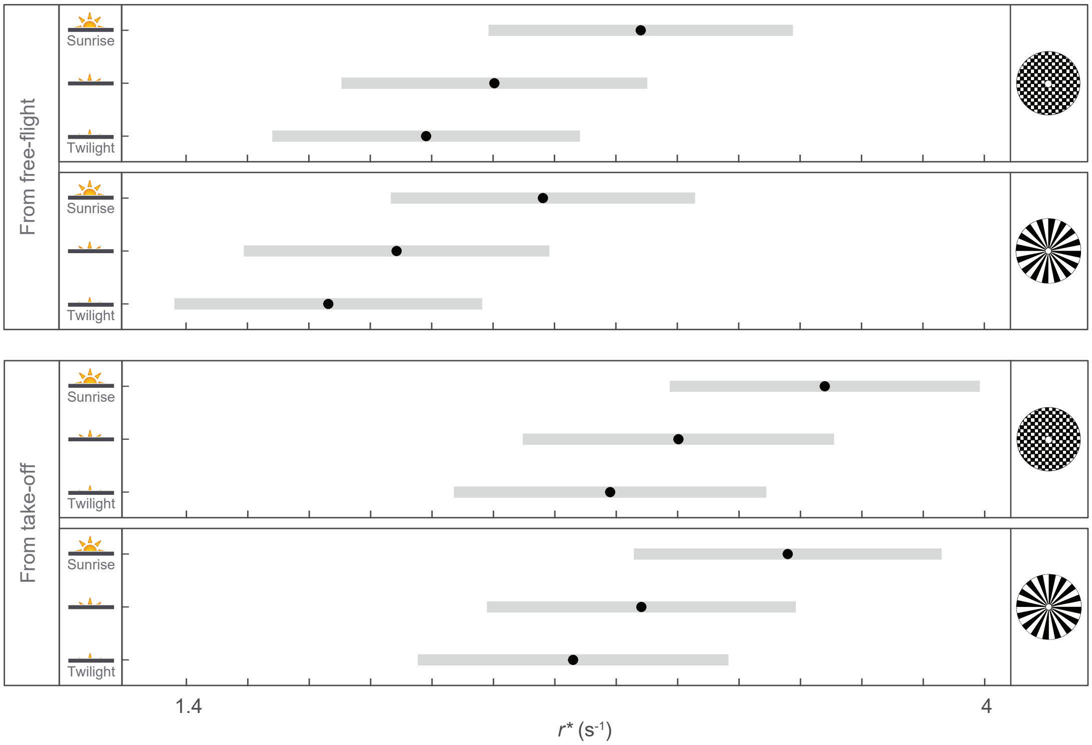

Figure S3: The mean relative-rate-of-expansion r^* as predicted by the linear mixed-effects model in three tested light conditions and two landing patterns for both landing types (from take-off and free-flight) using average-per-treatment analysis (related to Figure 3). The mean relative-rate-of-expansion increases with increase in light intensity and is higher when bumblebees initiate landing from take-off. It did not differ significantly between the two tested landing platforms (Table S2). Black dots depict estimated means and grey bars are 95% confidence intervals.

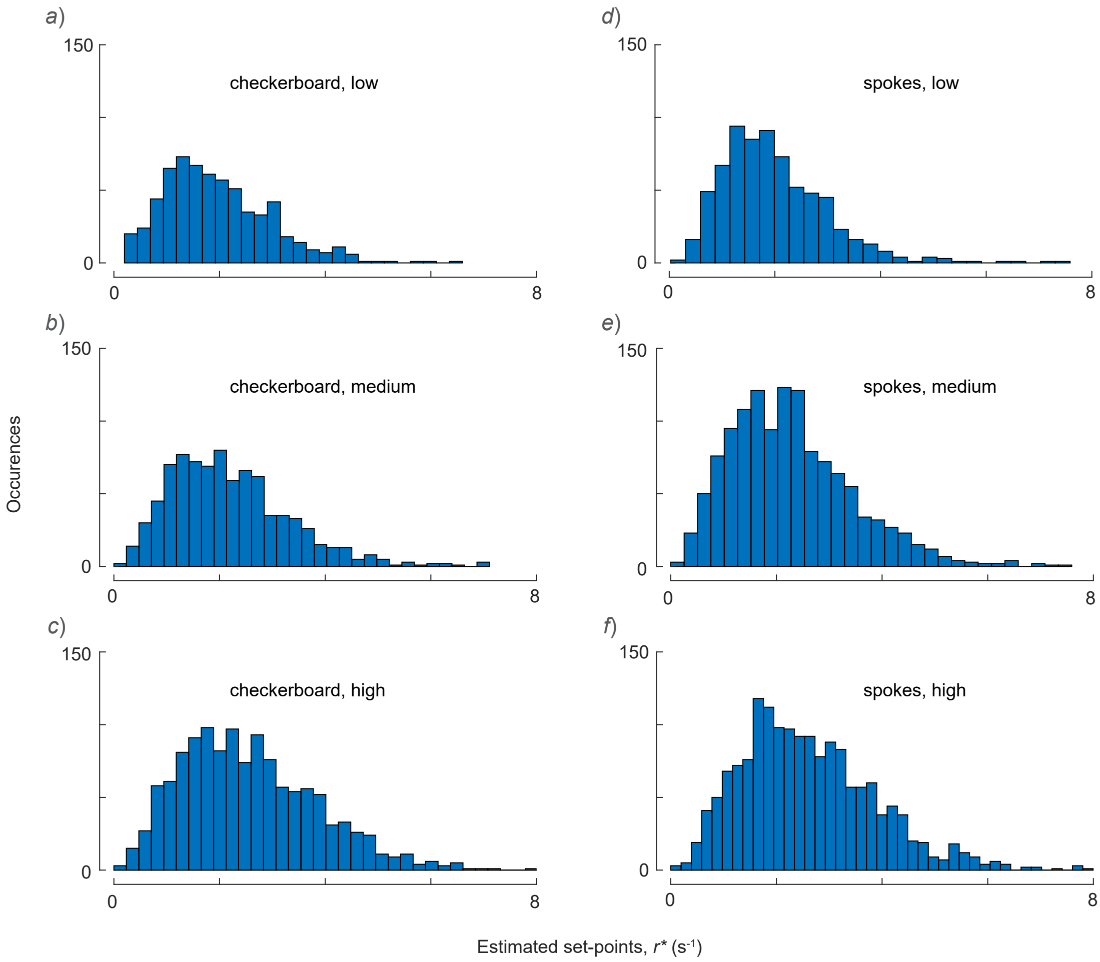

Figure S4: Histograms of the set-points of optical expansion rate r^* in all tested treatments (related to Figure 4). The conditions are: (a) checkerboard pattern, low light condition (n=643 segments), (b) checkerboard pattern, medium light condition (n=847 segments), (c) checkerboard pattern, high light condition (n=1243 segments), (d) spoke pattern, low light condition (n=700 segments), (e) spoke pattern, medium light condition (n=1255 segments), (f) spoke pattern, high light condition (n=1603 segments). (a-f) Each panel contains set-points for both landing types (landings initiated from take-off and free-flight).

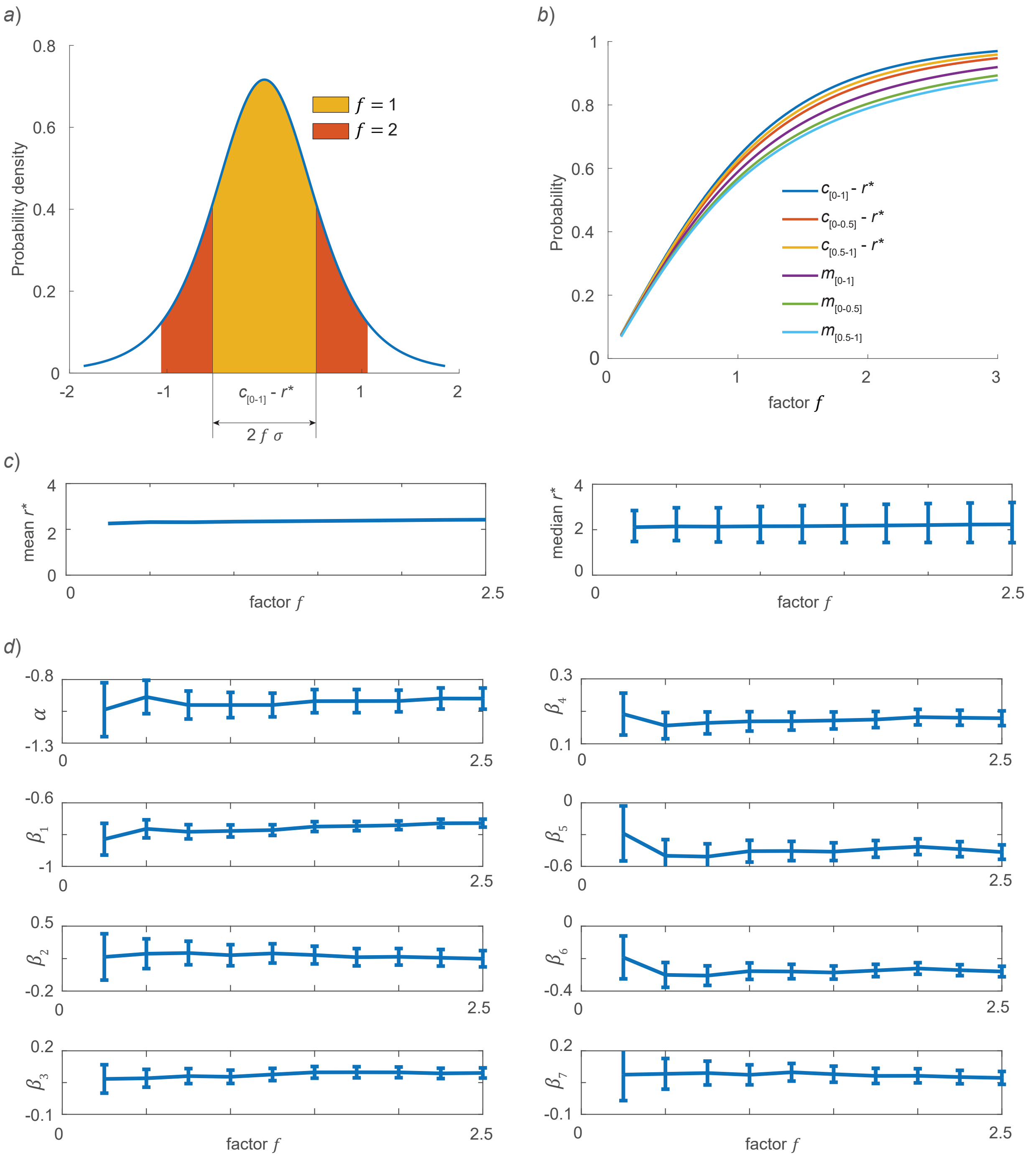

Figure S5: The effect of factor f on the results (related to Figures 4 – 6). (a) The threshold of variation allowed around the mean for $f = 1$ (yellow) and $f = 2$ (orange) for the probability density function of parameter $c_{[0-1]} - r^*$, $\sigma = 0.53$. (b) The probability of values that lie within f scale-parameter band around the mean of each parameter ($c_{[0-1]} - r^*$, $c_{[0-0.5]} - r^*$, $c_{[0.5-1]} - r^*$, $m_{[0-1]}$, $m_{[0-0.5]}$, and $m_{[0.5-1]}$). (c) The mean and median, 25 percentile and 75 percentile of the set-points of relative-rate-of-expansion identified at various values of the factor f. (d) The dependence of r^* on distance from the platform (y^*) along with the effect of different environmental conditions (landing patterns and light conditions) and landing type (take-off or free-flight) as per Equation S2 for each factor ($\log(r^*_{i,d,a,s}) \sim N(\alpha + \alpha_d + \alpha_a + \alpha_s + \beta_1 \ \log(y^*_{i,d,a,s}) + \beta_2 \ \mathrm{SPOKE}_{i,d,a,s} + \beta_3 \ \mathrm{MEDIUMlight}_{i,d,a,s} + \beta_4 \ \mathrm{HIGHlight}_{i,d,a,s} + \beta_5 \ \mathrm{fromTakeoff}_{i,d,a,s} + \beta_6 \ \log(y_{i,d,a,s}) \times \mathrm{fromTakeoff}_{i,d,a,s} + \beta_7 \ \log(y_{i,d,a,s}) \times \mathrm{SPOKE}_{i,d,a,s}, \ \sigma^2)$, vertical bars for each coefficient indicate 95% confidence intervals).

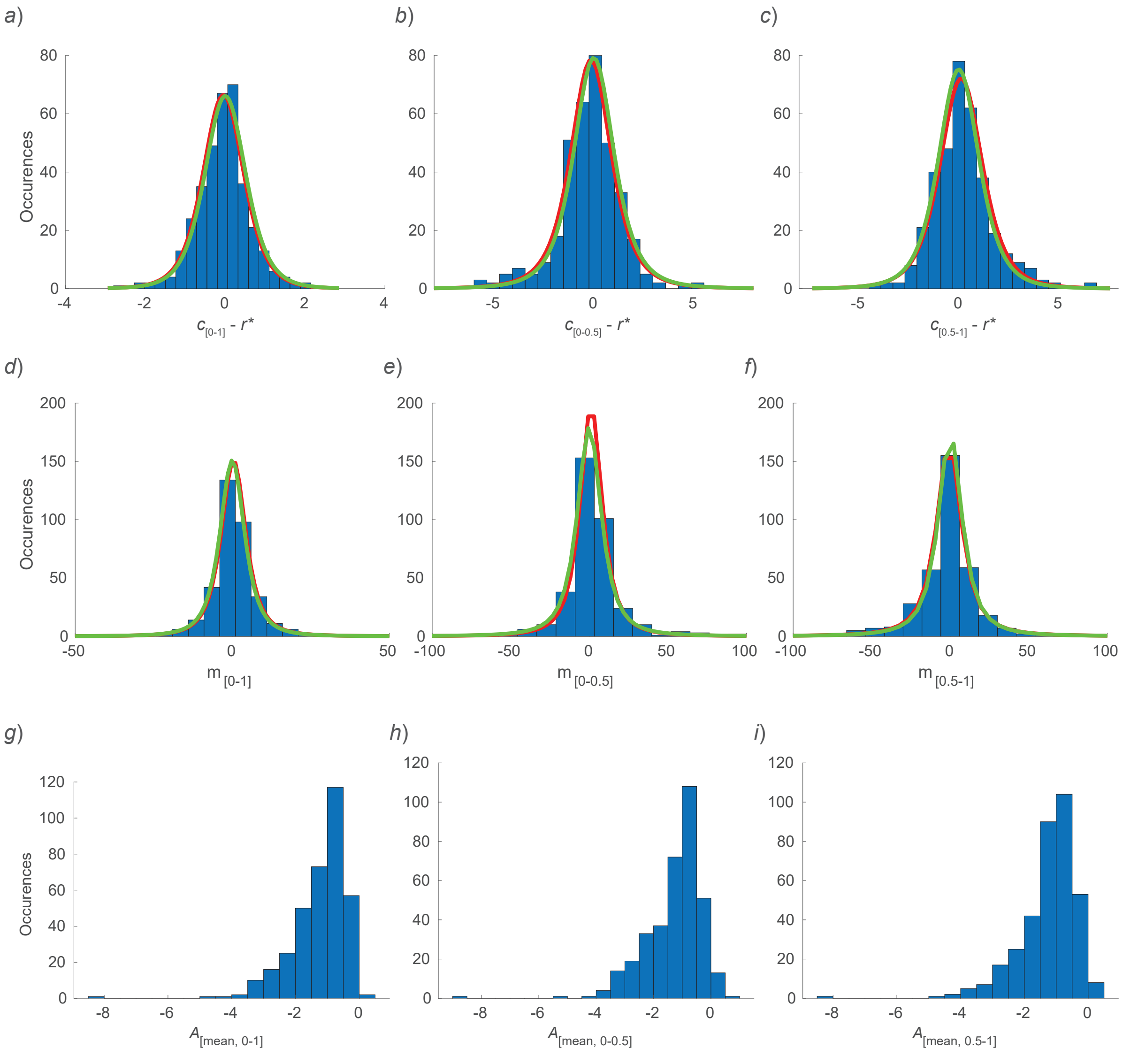

Figure S6: Distributions of nine parameters used in the constant-r extraction algorithm (related to Figures $4-6$). (a) $c_{[0-1]} - r^*$, (b) $c_{[0-0.5]} - r^*$, (c) $c_{[0.5-1]} - r^*$, (d) $m_{[0-1]}$, (e) $m_{[0-0.5]}$, (f) $m_{[0.5-1]}$, (g) $A_{[\text{mean},0-1]}$), (h) $A_{[\text{mean},0-0.5]}$, and (i) $A_{[\text{mean},0.5-1]}$. In blue, histograms of nine parameters obtained from manual selection of 355 constant-r segments in 313 (out of first 532) landing manoeuvres for high light condition and spoke landing pattern. In red, estimated probability density functions of first six parameters (generalized student's t-distributions, see Table S8 for parameter values). In green, the representative probability density functions of first six parameters used in constant-r extraction algorithm (location parameter μ for these six parameters is zero, scale parameter σ is σ_1, $2\sigma_1$, $2\sigma_1$, σ_2, $2\sigma_2$ and $2\sigma_2$ for panels (a)-(f), respectively (where $\sigma_1 = 0.53$ and $\sigma_2 = 4.22$) and shape parameter ν is same as in Table S8).

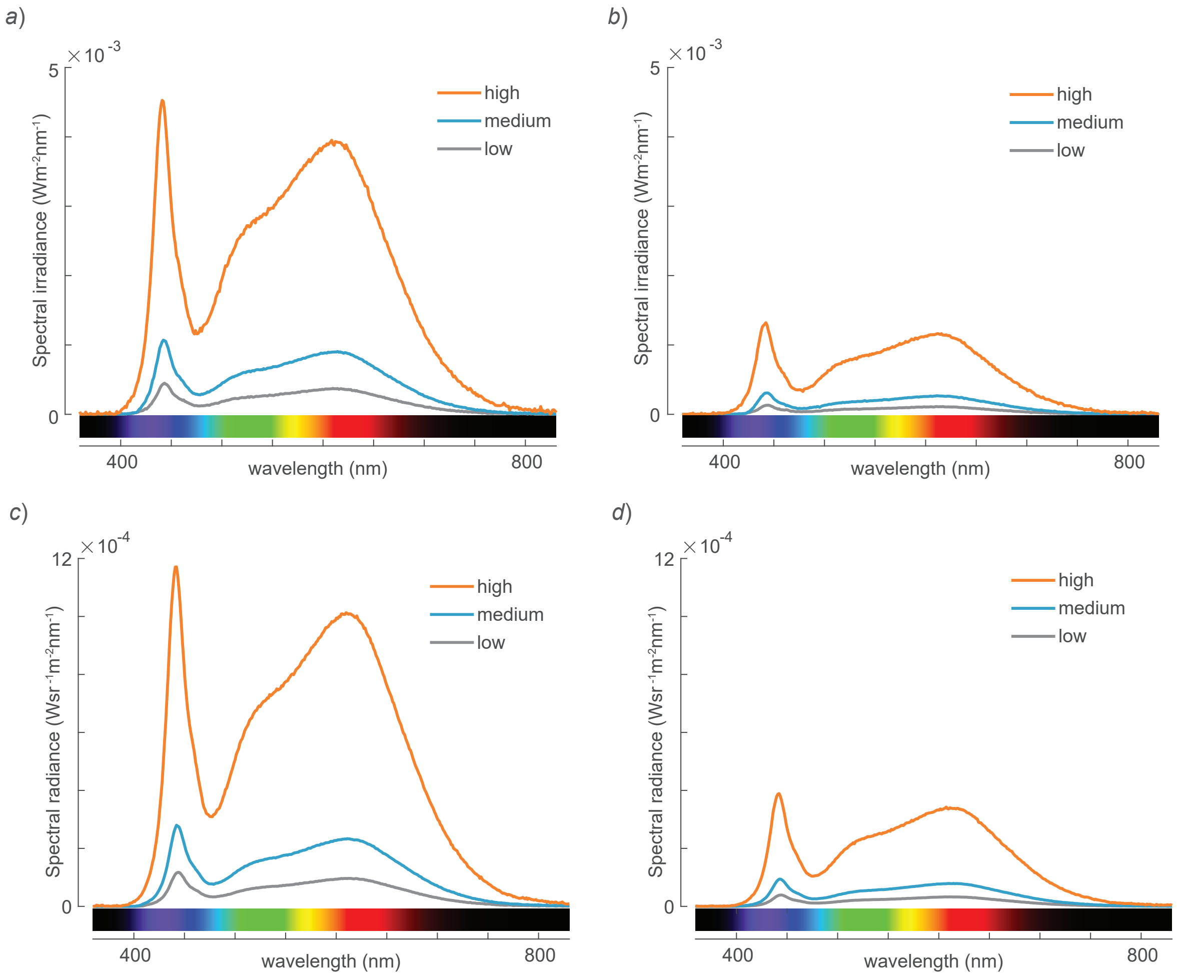

Figure S7: Light intensities for the low (grey), medium (blue) and high (orange) light conditions (related to Figure 2). Spectral irradiance (a) and spectral radiance (c) at the center of the flight arena. Spectral irradiance (b) and spectral radiance (d) at the centre of the landing platforms.

Table S1: The number of landing manoeuvres recorded in each tested treatment and the number of landing manoeuvres that are identified with constant-r segments for different values of factor f (related to Figure 4).

Treatment (landing pattern, light condition)	Number of landing manoeuvres	Factors f									
		0.25	0.5	0.75	1	1.25	1.5	1.75	2	2.25	2.5
From free-flight											
checkerboard, low	579	55	149	225	300	362	395	423	452	473	484
checkerboard, medium	923	71	221	358	472	545	605	649	687	719	744
checkerboard, high	1886	94	327	541	729	871	988	1099	1188	1271	1347
spoke, low	520	70	162	241	303	348	380	405	431	449	461
spoke, medium	1188	104	294	474	626	747	834	893	939	995	1027
spoke, high	2117	129	383	639	883	1065	1198	1325	1426	1521	1589
From take-off											
checkerboard, low	242	26	79	125	152	163	182	191	200	208	216
checkerboard, medium	287	24	67	96	137	169	189	203	219	230	238
checkerboard, high	554	35	99	164	229	271	312	343	377	406	422
spoke, low	298	30	88	123	166	196	224	234	250	266	271
spoke, medium	593	52	149	237	296	359	402	435	454	476	492
spoke, high	818	62	179	283	379	449	512	549	596	629	660
Total landings	10005	752	2197	3506	4672	5545	6221	6749	7219	7643	7951

Table S2: Analysis of mean relative-rate-of-expansion in different tested treatments (landing patterns, light conditions and starting conditions) for average-per-treatment analysis method (related to Figure 3). The data comprises of 10,005 landing approaches between 0.04 m $\leq y \leq 0.11$ m, where y is the perpendicular distance to the platforms. Post-hoc tests compare differences between mean relative-rate-of-expansion observed in different tested conditions (statistical model as given by Equation S1: $r_{i,d,a,s} \sim N(\alpha + \alpha_d + \alpha_a + \alpha_s + \beta_1$ SPOKE$_{i,d,a,s} + \beta_2$ MEDIUMlight$_{i,d,a,s} + \beta_3$ HIGHlight$_{i,d,a,s}$ + β_4 fromTakeoff$_{i,d,a,s} + \beta_5$ SPOKE$_{i,d,a,s} \times$ fromTakeoff$_{i,d,a,s}$, σ^2)).

Fixed effect	Estimate	Std error	t value	Pr(>|t|)
α	2.18	0.26	8.51	0.034
β_1	-0.32	0.15	-2.17	0.056
β_2	0.22	0.05	4.07	4.67E-5
β_3	0.70	0.05	13.90	< 2E-16
β_4	0.60	0.06	9.64	< 2E-16
β_5	0.20	0.08	2.44	0.015

Post-hoc contrasts*	Estimate	Std error	z ratio	p value
(H + F) - (L + F)	0.70	0.05	13.90	4.1E-42
(H + F) - (M + F)	0.48	0.04	11.76	3.95E-30
(H + F) - (H x F)	0.32	0.15	2.17	1
(H + F) - (L x F)	1.02	0.16	6.53	4.3E-09
(H + F) - (M x F)	0.80	0.15	5.21	1.24E-05
(L + F) - (M + F)	-0.22	0.05	-4.07	0.003054
(L + F) - (H x F)	-0.38	0.16	-2.45	0.930313
(L + F) - (L x F)	0.32	0.15	2.17	1
(L + F) - (M x F)	0.10	0.16	0.61	1
(M + F) - (H x F)	-0.16	0.15	-1.04	1
(M + F) - (L x F)	0.54	0.16	3.44	0.038107
(M + F) - (M x F)	0.32	0.15	2.17	1
(H x F) - (L x F)	0.70	0.05	13.90	4.1E-42
(H x F) - (M x F)	0.48	0.04	11.76	3.95E-30
(L x F) - (M x F)	-0.22	0.05	-4.07	0.003054
(H + T) - (L + T)	0.70	0.05	13.90	4.1E-42
(H + T) - (M + T)	0.48	0.04	11.76	3.95E-30
(H + T) - (H x T)	0.12	0.16	0.76	1
(H + T) - (L x T)	0.82	0.17	4.91	6.12E-05
(H + T) - (M x T)	0.60	0.16	3.65	0.017404
(L + T) - (M + T)	-0.22	0.05	-4.07	0.003054
(L + T) - (H x T)	-0.58	0.17	-3.48	0.033282
(L + T) - (L x T)	0.12	0.16	0.76	1
(L + T) - (M x T)	-0.10	0.17	-0.61	1
(M + T) - (H x T)	-0.36	0.16	-2.17	1
(M + T) - (L x T)	0.34	0.17	2.03	1
(M + T) - (M x T)	0.12	0.16	0.76	1
(H x T) - (L x T)	0.70	0.05	13.90	4.1E-42
(H x T) - (M x T)	0.48	0.04	11.76	3.95E-30
(L x T) - (M x T)	-0.22	0.05	-4.07	0.003054

*Low (L), medium (M) and high (H) light conditions, checkerboard (+) and spoke (x) landing patterns, free-flight (F) and take-off (T) starting conditions.

*Comparisons among starting conditions also formed part of post-hoc test, but are not shown here.

Table S3: Number of landing manoeuvres that contain more than one set-points of relative-rate-of-expansion along with different characteristics (Δr^*, Δy_1 and Δy_2, related to Figure 4 and 5) in different tested treatments (factor $f = 1$).

Treatment (pattern, light)	No. of landing tracks with constant-r segments	No. of constant-r segments	No. of manoeuvres with >1 constant-r segments	For 1298 manoeuvres with more than one constant-r segments				For 1015 manoeuvres with two constant-r segments	
				No. of constant-r segments	No. of jumps to a different r^*	For jumps to a higher r^*: % of jumps	For jumps to a higher r^*: $\Delta r^*(s^{-1})$ (mean [std-dev.])	$\Delta y_1(m)$ (mean [std-dev.])	$\Delta y_2(m)$ (mean [std-dev.])
From free-flight									
L+	300	418	94	212	118	73.7%	0.740 [0.688]	0.030 [0.015]	0.099 [0.040]
M+	472	667	148	343	195	72.3%	1.023 [0.964]	0.033 [0.016]	0.110 [0.044]
H+	729	939	175	385	210	74.8%	1.394 [1.120]	0.037 [0.018]	0.116 [0.053]
Lx	303	454	118	269	151	67.6%	0.778 [0.595]	0.030 [0.015]	0.107 [0.046]
Mx	626	854	180	408	228	71.5%	1.047 [0.846]	0.034 [0.018]	0.111 [0.047]
Hx	883	1103	187	407	220	75.0%	1.198 [0.964]	0.036 [0.017]	0.112 [0.047]
From take-off									
L+	152	225	54	127	73	75.3%	0.986 [0.873]	0.033 [0.018]	0.132 [0.048]
M+	137	180	37	80	43	74.4%	1.052 [1.065]	0.036 [0.019]	0.121 [0.060]
H+	229	304	64	139	75	73.3%	1.305 [1.184]	0.037 [0.017]	0.114 [0.052]
Lx	166	246	61	141	80	75.0%	0.854 [0.733]	0.035 [0.019]	0.120 [0.052]
Mx	296	401	82	187	105	72.4%	0.967 [0.892]	0.036 [0.017]	0.122 [0.053]
Hx	379	500	98	219	121	65.3%	1.221 [1.020]	0.039 [0.020]	0.128 [0.054]

Low (L), medium (M) and high (H) light conditions, checkerboard (+) and spoke (x) landing patterns.

Table S4: Analysis of relative-rate-of-expansion set-points (r^*) dependence on distance to the platform (y^*) in different tested treatments (landing patterns and light conditions) and with different starting conditions (take-off and free-flight) for per-track analysis method (related to Figure 5). The data comprises of r^* and y^* for 2,917 constant-r segments in 1,298 landing manoeuvres that contain more than one constant-r segments (factor $f = 1$) (statistical model as given by Equation S2: $\log(r^*_{i,d,a,s}) \sim N(\alpha + \alpha_d + \alpha_a + \alpha_s + \beta_1\ \log(y^*_{i,d,a,s}) + \beta_2\ \mathrm{SPOKE}_{i,d,a,s} + \beta_3\ \mathrm{MEDIUMlight}_{i,d,a,s} + \beta_4\ \mathrm{HIGHlight}_{i,d,a,s} + \beta_5\ \mathrm{fromTakeoff}_{i,d,a,s} + \beta_6\ \log(y_{i,d,a,s}) \times \mathrm{fromTakeoff}_{i,d,a,s} + \beta_7\ \log(y_{i,d,a,s}) \times \mathrm{SPOKE}_{i,d,a,s},\ \sigma^2))$.

Fixed effect	Estimate	Std error	t value	Pr(>|t|)
α	-0.98	0.06	-17.64	1.11E-47
β_1	-0.74	0.03	-28.88	5.3E-161
β_2	0.18	0.07	2.62	0.009372
β_3	0.06	0.02	2.85	0.004463
β_4	0.17	0.02	8.00	2.68E-15
β_5	-0.26	0.07	-3.76	0.000173
β_6	-0.16	0.03	-4.55	5.54E-06
β_7	0.09	0.03	2.65	0.008201

Table S5: Analysis of relative-rate-of-expansion set-points (r^*) dependence on distance to the platform (y^*) in different tested treatments (landing patterns and light conditions) and with different starting conditions (take-off and free-flight) for per-track analysis method (related to Figure 6). The data comprises of r^* and y^* for 6,291 constant-r segments in 4,672 landing manoeuvres. Post-hoc tests compare differences in $log(r^*)$ observed at mean $y^* = 0.15m$ in the presence of different light conditions and landing platforms (factor $f = 1$) (statistical model as given by Equation S2: $\log(r^*_{i,d,a,s}) \sim N(\alpha + \alpha_d + \alpha_a + \alpha_s + \beta_1 \ \log(y^*_{i,d,a,s}) + \beta_2 \ \text{SPOKE}_{i,d,a,s} + \beta_3 \ \text{MEDIUMlight}_{i,d,a,s} + \beta_4 \ \text{HIGHlight}_{i,d,a,s} + \beta_5 \ \text{fromTakeoff}_{i,d,a,s} + \beta_6 \ \log(y_{i,d,a,s}) \times \text{fromTakeoff}_{i,d,a,s} + \beta_7 \ \log(y_{i,d,a,s}) \times \text{SPOKE}_{i,d,a,s}, \ \sigma^2))$.

Fixed effect	Estimate	Std error	t value	Pr(>|t|)
α	-1.00	0.05	-20.13	1.53E-17
β_1	-0.78	0.02	-40.76	0
β_2	0.19	0.06	3.28	0.001397
β_3	0.08	0.02	4.92	8.81E-07
β_4	0.17	0.01	11.31	3.31E-29
β_5	-0.46	0.05	-8.85	1.09E-18
β_6	-0.28	0.03	-10.93	1.44E-27
β_7	0.09	0.02	3.67	0.000248

Post-hoc constrasts* in $log(r^*)$ at mean $y^* = 0.15m$	Estimate	Std error	z ratio	p value
L F - M F	-0.08	0.02	-4.92	1.27E-05
L F - H F	-0.17	0.01	-11.31	1.72E-28
L F - L T	-0.07	0.01	-5.58	3.68E-07
L F - M T	-0.15	0.02	-7.13	1.53E-11
L F - H T	-0.24	0.02	-11.94	1.14E-31
M F - H F	-0.09	0.01	-7.17	1.1E-11
M F - L T	0.01	0.02	0.35	1
M F - M T	-0.07	0.01	-5.58	3.68E-07
M F - H T	-0.16	0.02	-9.08	1.63E-18
H F - L T	0.10	0.02	5.17	3.46E-06
H F - M T	0.02	0.02	1.17	1
H F - H T	-0.07	0.01	-5.58	3.68E-07
L T - M T	-0.08	0.02	-4.92	1.27E-05
L T - H T	-0.17	0.01	-11.31	1.72E-28
M T - H T	-0.09	0.01	-7.17	1.1E-11

*Low (L), medium (M) and high (H) light conditions, free-flight (F) and take-off (T) starting conditions.

*The results are averaged over patterns because comparisons of r^* among landing patterns for each light condition and starting condition were similar.

Table S6: Analysis of relative-rate-of-expansion set-points (r^*) dependence on distance to the platform (y^*) in different tested treatments (landing patterns and light conditions) and with different starting conditions (take-off and free-flight) for per-track analysis method (related to Figures 5 and 6). The data comprises of r^* and y^* for 3,374 constant-r segments in 3,374 landing manoeuvres, with one constant-r segment in each manoeuvre (factor $f = 1$) (statistical model as given by Equation S2: $\log(r^*_{i,d,a,s}) \sim N(\alpha + \alpha_d + \alpha_a + \alpha_s + \beta_1 \ \log(y^*_{i,d,a,s}) + \beta_2 \ \mathrm{SPOKE}_{i,d,a,s} + \beta_3 \ \mathrm{MEDIUMlight}_{i,d,a,s} + \beta_4 \ \mathrm{HIGHlight}_{i,d,a,s} + \beta_5 \ \mathrm{fromTakeoff}_{i,d,a,s} + \beta_6 \ \log(y_{i,d,a,s}) \times \mathrm{fromTakeoff}_{i,d,a,s} + \beta_7 \ \log(y_{i,d,a,s}) \times \mathrm{SPOKE}_{i,d,a,s}, \ \sigma^2))$.

Fixed effect	Estimate	Std error	t value	Pr(>|t|)
α	-0.97	0.07	-14.15	2.68E-17
β_1	-0.79	0.03	-28.56	8.2E-161
β_2	0.22	0.08	2.82	0.005261
β_3	0.08	0.02	3.49	0.000484
β_4	0.14	0.02	6.96	4.2E-12
β_5	-0.67	0.07	-8.99	4.16E-19
β_6	-0.40	0.04	-11.02	8.71E-28
β_7	0.10	0.03	3.01	0.002627

Table S7: Pseudo-random treatment schedule followed during experiments (related to Figure 2).

Time (hours)		Days													
start	end	1	2	3	4	5	6	7	8	9	10	11	12	13	14
80000	93000	M+	Lx	L+	Hx	L+	Hx	H+	Mx	H+	Mx	M+	Lx	M+	Lx
93000	110000	H+	Mx	H+	Mx	M+	Lx	M+	Lx	L+	Hx	L+	Hx	H+	Mx
110000	123000	L+	Hx	M+	Lx	H+	Mx	L+	Hx	M+	Lx	H+	Mx	L+	Hx
123000	140000	M+	Lx	L+	Hx	L+	Hx	H+	Mx	H+	Mx	M+	Lx	M+	Lx
140000	153000	H+	Mx	H+	Mx	M+	Lx	M+	Lx	L+	Hx	L+	Hx	H+	Mx
153000	170000	L+	Hx	M+	Lx	H+	Mx	L+	Hx	M+	Lx	H+	Mx	L+	Hx

Low (L), medium (M) and high (H) light conditions, checkerboard (+) and spoke (x) landing patterns.

Table S8: Estimated location parameter μ, scale parameter σ and shape parameter ν for six variables that together define the constancy of relative-rate-of-expansion in a track segment (values are mean [95% confidence intervals]) (related to Figures 4 and S6).

Variables	μ	σ	ν
$c_{[0-1]} - r^*$	-0.05 [-0.11, 0.02]	0.53 [0.46, 0.60]	5.02 [2.87, 8.77]
$c_{[0-0.5]} - r^*$	-0.14 [-0.28, -0.00]	1.07 [0.93, 1.23]	3.24 [2.20, 4.76]
$c_{[0.5-1]} - r^*$	0.12 [-0.02, 0.26]	1.11 [0.96, 1.27]	3.92 [2.47, 6.23]
$m_{[0-1]}$	0.39 [-0.16, 0.95]	4.22 [3.63, 4.91]	2.30 [1.69, 3.13]
$m_{[0-0.5]}$	1.20 [0.16, 2.24]	7.67 [6.57, 8.95]	1.82 [1.41, 2.35]
$m_{[0.5-1]}$	-0.72 [-1.93, 0.49]	8.85 [7.47, 10.48]	1.64 [1.27, 2.12]

S1. Transparent methods

S1.1. Experimental Animals

We used a commercially available hive of bumblebees (*Bombus terrestris*) from Koppert B.V. (Berkel en Rodenrijs, the Netherlands) for our experiments. The colony contained 50–70 worker bumblebees (female) that engaged in different activities required to maintain the hive. Among others, they performed foraging flights to collect artificial nectar (50% sugar solution obtained from Koppert B.V.) from a feeding platform. They had ad libitum access to the food source during the day and were given dried pollen directly in the hive at the end of each day. We placed the hive in an indoor laboratory where the temperature was maintained at 21 ± 2°C and the hive remained connected to our setup for the entire duration of the experiment.

S1.2. Experimental setup

The experimental setup consisted of a flight arena (3 × 0.48 × 0.48 m; length×width×height), with a bumblebee hive, food source, and a real-time machine-vision based videography system for tracking the flying bumblebees (Figure 2a,b). The top, bottom, and longitudinal side walls of the flight arena were made of transparent poly-carbonate sheets (thickness 0.01 m) and the far ends were closed with meshes. We installed the hive and a food source (containing sugar solution) outside the flight chamber and directly opposite to each other (Figure 2a). The hive and the food-source were connected to the longitudinal walls of the flight chamber near its middle section using Plexiglas tubes. These tubes extended 0.07 m inside the flight chamber and had vertical landing platforms attached at the end (Figure 2b). The landing discs were covered with either a checkerboard pattern (0.01 m black and white squares) or spoke pattern (32 spokes filled with alternating black and white colours) (Figure 2c) printed on a normal paper.

The flight chamber was illuminated with white broad-spectrum LED light panel (Lumihome 595×595 mm, 4800 lm, LED type: SMD2835, 4000K), powered with 300W power supply (maximum 40 V and 1.1 A) (RS PRO RS6005P). This light panel was placed 50 cm above the chamber's top wall and right above the middle section of the flight arena (Figure 2a,b). The light panel could be set to three different light intensities by PWM dimming: a low light condition simulating dusk (13.7 lx), a medium light condition (33.3 lx), and a high light condition equivalent to sunrise (144.9 lx). Reported light intensities were the average at the centre of the flight arena and at the landing platforms, measured using a spectroradiometer (Specbos 1211 with JETI Lival software, Figure S7).

Flight movements of the foraging bumblebees were tracked in 3D using a real-time machine-vision based videography system. The system consisted of four synchronized high-speed cameras (one Basler acA1300-200um camera with 12 mm Fujinon lens and three Basler acA2040-90um cameras with 12.5 mm Kowa lenses), which viewed the central portion of the arena from two orthogonal positions above the arena and two orthogonal positions from the side (Figure 2a). The cameras operated at 175 frames per second, image resolution was 504×504 pixels for Basler acA2040-90um cameras and 512×512 pixels for Basler acA1300-200um camera, and image depth was 8 bits. Exposure times were 260 µs, 200 µs, 400 µs and 900 µs for top-left (Basler acA2040-90um), top-right (Basler acA2040-90um), side-left (Basler acA2040-90um), and side-right (Basler acA1300-200um) cameras, respectively. All cameras were back-lit using custom-built arrays of infrared (IR) LED panels (centroid wavelength: 850 nm) to enhance the contrast of bumblebees with the background (19.25 V, 1 A).

When running, the real-time machine-vision based videography system estimated the three-dimensional position of all moving bumblebees at each recording time step. Based on these, movement trajectories for each bumblebee were constructed. To remove tracking noise from these trajectories, we filtered these using a low-pass second-order two-directional Butterworth filter (*filtfilt* in Matlab 2020a, MathWorks Inc) with a cut-off frequency of 20 Hz, and then stored in arrays with space-time vectors $\mathbf{X}_G = (x_G, y_G, z_G, t_G)$. Position was defined in the global coordinate system (not shown), and time resolution equalled that of the videography system (1/175 s). The software also had the option to automatically store video images to disc, based on tracking output. We calibrated the videography system four times throughout the experiments using Direct Linear Transformation; a correction for lens distortion was performed once (Svoboda, Martinec and Pajdla, 2005; Straw, Branson, Neumann and Dickinson, 2011).

S1.3. Experimental procedure

Before starting the experiments, we trained the bumblebees for 10 days to forage in the arena at the low light intensity condition. At the start of the training period, we directly connected the hive and food source with a bridge, allowing the bumblebees to walk to the food source; each day, we gradually increased the bridge gap until after 10 days the forager bumblebees would comfortably fly back and forth between hive and food source.

During training and experiments, we exposed bumblebees to a day-night cycle of 10-14 hours with day starting at 07:30h and ending at 17:30h. Sunrise (07:30h–08:00h) was simulated by gradually increasing the light intensity from zero to the light condition for the experiment (low, medium or high) or training (low). Similarly, sunset (17:00h–17:30h) was simulated by gradually reducing the last light condition of the day down to zero. During experiments, the rest of the day (08:00h–17:00h) was divided into 6 time-slots of 1.5 hours each. We changed the light condition in each time-slot and landing platform every day (Table S7).

Since the recorded landing manoeuvres are likely to be highly stereotypical, we used a single hive in our study. The recorded landing manoeuvres are thus pseudo-replications of foraging bumblebees that existed in the hive over a span of 14 consecutive days.

S1.4. Extraction of landing tracks

From all trajectory data of bumblebees inside the flight arena, we first selected the tracks in which bumblebees were flying, as tracks that were less than 5% of the time closer than 6 cm from the side walls or less than 0.1% of the time closer than 5 cm from the top and bottom surfaces. Among the resulting flight tracks, we then selected the tracks in which bumblebees approached one of the landing platforms. These landing approach tracks were defined as tracks that started at a normal distance (along y-axis) of at least 10 cm from the landing platform, and had a minimum normal distance to the platform of less than 2 cm. For each track that met those criteria, the landing approach manoeuvre was selected as the trajectory section between the distance furthest away from the landing platform and closest distance (up to 1cm). Furthermore, the tracks starting close to either of the platforms (within 2.5 cm long cylinder around opposite disc) or the ground (less than 2 cm distance from ground) were labelled as the landing approaches starting from a take-off and the rest of the tracks were labelled as the landing approaches from free-flight condition.

The landing approaches were stored as space-time arrays $\mathbf{X} = (x, y, z, t)$, in the landing platform coordinate system (Figure 2c). This cartesian coordinate system has its origin at the centre of the landing platform, y normal to the platform, and z upwards. Time t was set to zero at the end of the trajectory, i.e. when the bumblebee reached the closest distance to the platform. The corresponding velocity and acceleration vectors ($\mathbf{U} = (u, v, w)$ and $\mathbf{A} = (a_x, a_y, a_z)$, respectively) were computed by numerical differentiation using a second-order central differentiation scheme and stored. These approach sequences, referred to as landing manoeuvres, were analysed in this study.

S1.5. Estimation of state variables and set-points of relative-rate-of-expansion (r^*)

To analyse the landing dynamics of bumblebees, we focused on the movement normal to the landing platform, which can be described by the state variables: normal distance from the platform $y(t)$, flight velocity towards the platform $V(t) = -v(t)$, and acceleration towards the platform $A(t) = -a_y(t)$. Based on these, we calculated the instantaneous relative rate of optical expansion throughout each landing approach track, as $r(t) = V(t)/y(t)$. In total, we thus used the temporal dynamics of four state variables (y, V, A, r) to describe the dynamics of bumblebees decelerating as they approach a surface for landing. A previous study suggested that bumblebees on an average hold the relative-rate-of-expansion constant during a landing approach (Chang, Crall and Combes, 2016). Therefore, we developed a custom-made search algorithm for automatic identification of the landing approach segments in which the relative-rate-of-expansion was close to a constant as bumblebees were decelerating.

The constant-r detection algorithm is based on six parameters that together define the constancy of r in a track segment. For an arbitrary track segment, these six parameters are computed from three first-order linear regressions (one regression in full segment, and two regressions in two equal halves of the full segment) and they evaluate the deviations of these regressions from a constant r regression. To evaluate these deviations, we first find the expected probability distributions for each of these six parameters when bumblebees were flying with constant r, and then check whether the six parameters computed for an arbitrary segment lie within certain threshold around the expected mean of each parameter. This threshold is specified by a setting parameter f whose value can be changed to alter the variation allowed around the mean of each of the six linear regression parameters for an arbitrary segment to be identified as a constant-r segment. This f is thus similar to the number of standard deviations around the mean of a normally distributed variable that is included in the selection. Hence, by finding these parameters for different track segments in a landing manoeuvre, we could identify all track segments in which a bumblebee kept the variation of r below a threshold defined by a fixed value of factor f.

The identified track segments are called constant-r segments and are characterized by their average values of the four state variables (y^*, V^*, A^*, r^*). Here, r^* is a linear regression estimate from $r(t) = r^* + \epsilon$ (where ϵ denotes residuals) within a constant-r segment, and we use it as an estimate of the set-point of relative-rate-of-expansion that

the bumblebee aims to hold constant. This is because r^* for each constant-r track segment is very close to m, where m is a linear regression estimate of $V(t) = m \times y(t) + \epsilon$ (ϵ denotes residuals) in a constant-r segment (mean, median and maximum difference between the two values are 0.005, 0.002 and 0.096 s^{-1}, respectively). Moreover, the difference between the actual flight duration and the analytically computed flight duration if the bumblebees had followed exactly the estimated set-points of relative-rate-of-expansion within identified constant-r segments is extremely low (maximum difference = 0.0086 s which is only 1.5 times 1/175 s, the time resolution of the experimental apparatus). Additionally, we only consider the velocity perpendicular to the platforms as this component of the velocity was needed to be progressively built and later progressively reduced as bumblebees approached the platforms for landing. However, the deceleration strategy of adjusting these set-points with distance remains unchanged if 3D speed is considered.

S1.6. Statistical models

All statistical analyses were done in R 4.0.2 (R Foundation). We used $lmer$ to develop different linear mixed-effects models for the two analysis methods. The landing patterns, light conditions, landing type (take-off and free-flight) along with their (significant) interactions are considered as fixed factors, and day of the experiment, landing side (whether the landing disc is located towards the hive or the food-source) and each landing approach are considered as random intercepts. We used model dredging to identify the minimal linear mixed-effects statistical model. For post-hoc tests, we used Bonferroni correction (using *emmeans* package in R) to adjust the statistical significance values for comparison of means and covariates in different treatments (landing patterns and light conditions) and for different landing types. p-values < 0.05 were considered statistically significant. Unless stated otherwise, data-sets averages and distributions are given as mean±standard deviation, including sample size (n); statistical model predictions are given as mean [standard error], including p-values if relevant.

Specifically, the linear mixed-effects models were developed to determine (a) the set-points of relative-rate-of-expansion (r^*) in average-per-treatment method, (b) the set-point variation with distance from the landing platforms (y^*) in per-track method, and (c) the effect of landing patterns, light conditions and landing type (landings starting from take-off or free-flight) in both analyses.

S1.6.1. The average-per-treatment model

For the average-per-treatment analysis method, we computed the mean relative-rate-of-expansion in each tested treatment by first using pattern, light, starting conditions along with all possible interactions as fixed factors and day of the experiment, landing approach number, and landing side (whether landing disc is located on the hive side or the food source side) as random factors. The model dredging revealed only $pattern \times startingCondition$ as significant, therefore we used the reduced model (Equation S1).

$$\begin{aligned} r_{i,d,a,s} \sim N(\alpha + \alpha_d + \alpha_a + \alpha_s + \beta_1\ \mathrm{SPOKE}_{i,d,a,s} + \beta_2\ \mathrm{MEDIUMlight}_{i,d,a,s} + \beta_3\ \mathrm{HIGHlight}_{i,d,a,s} + \\ \beta_4\ \mathrm{fromTakeoff}_{i,d,a,s} + \beta_5\ \mathrm{SPOKE}_{i,d,a,s} \times \mathrm{fromTakeoff}_{i,d,a,s},\ \sigma^2) \end{aligned} \tag{S1}$$

where $r_{i,d,a,s}$ is the relative-rate-of-expansion for the i-th measurement from d-th day ($d \in \{1, 2, \dots, 14\}$), a-th landing approach ($a \in \{1, 2, \dots, 10005\}$) and s-th landing side ($s = 1$ for hive side and $s = 2$ for food-source side), α is the regression intercept for checkerboard pattern and low light condition (overall intercept), α_d is the day-specific intercept, α_a is the landing-approach-specific intercept, α_s is the landing-side-specific intercept, $\mathrm{SPOKE}_{i,d,a,s}$, $\mathrm{MEDIUMlight}_{i,d,a,s}$, $\mathrm{HIGHlight}_{i,d,a,s}$ and $\mathrm{fromTakeoff}_{i,d,a,s}$ indicate if spoke landing pattern, medium light condition, high light condition and take-off are present for the i-th measurement from d-th day, a-th landing approach and s-th landing side (0 = no, 1 = yes), $\beta_i\ \forall i \in \{1, 2, 3, 4, 5\}$ represent differences of fixed-effects and an interaction term from overall intercept, and σ is the residual standard deviation. The statistical output, along with post-hoc tests, from data of 10,005 landing approaches in the selected range of distance to the platforms ($0.04m \le y \le 0.11m$) is given in Table S2.

S1.6.2. The per-track analysis model

For the per-track analysis method, the dependence of set-points of relative-rate-of-expansion (r^*) on distance to the platform (y^*) was deemed based on the interdependence of r and y for a constant-$\dot{\tau}$ landing strategy (Equation S8b) i.e., a linear relationship between their log transformations. To adjudge the dependence of r^* on y^* as per constant-$\dot{\tau}$ law, we first constructed a full model with $\log(r^*)$ as response variable, $\log(y^*)$, landing patterns, light conditions, landing types along with all interactions as fixed factors, and day of the experiment, landing approach and landing side (whether landing disc is located on the hive side or the food source side) as random intercepts. Among all interaction terms,

the model dredging revealed only $\log(y^*) \times pattern$ and $\log(y^*) \times startingCondition$ interaction terms as significant, therefore we used the following reduced model:

$$\begin{aligned}\log(r^*_{i,d,a,s}) \sim N(\alpha + \alpha_d + \alpha_a + \alpha_s + \beta_1\ \log(y^*_{i,d,a,s}) + \beta_2\ \text{SPOKE}_{i,d,a,s} + \beta_3\ \text{MEDIUMlight}_{i,d,a,s} + \\ \beta_4\ \text{HIGHlight}_{i,d,a,s} + \beta_5\ \text{fromTakeoff}_{i,d,a,s} + \beta_6\ \log(y_{i,d,a,s}) \times \text{fromTakeoff}_{i,d,a,s} + \\ \beta_7\ \log(y_{i,d,a,s}) \times \text{SPOKE}_{i,d,a,s},\ \sigma^2)\end{aligned} \quad \text{(S2)}$$

where $r^*_{i,d,a,s}$ and $y^*_{i,d,a,s}$ are set-point of relative-rate-of-expansion and mean distance, respectively, for the i-th constant-r segment from d-th day ($d \in \{1, 2, \ldots, 14\}$), a-th landing approach ($a \in \{1, 2, \ldots, 4672\}$) and s-th landing side ($s = 1$ for hive side and $s = 2$ for food-source side), α is the regression intercept for checkerboard pattern, low light condition and free-flight starting condition (overall intercept), α_d is the day-specific intercept, α_a is the landing-approach-specific intercept, α_s is the landing-side-specific intercept, $\text{SPOKE}_{i,d,a,s}$, $\text{MEDIUMlight}_{i,d,a,s}$, $\text{HIGHlight}_{i,d,a,s}$ and $\text{fromTakeoff}_{i,d,a,s}$ indicate if spoke landing pattern, medium light condition, high light condition and take-off starting condition are present for the i-th measurement from d-th day, a-th landing approach and s-th landing side (0 = no, 1 = yes), β_1 represents the regression slope for predictor $\log(y^*)$ (overall slope), $\beta_i\ \forall i \in \{2, 3, 4, 5, 6, 7\}$ represent differences of fixed-effects including an interaction from overall intercept and slope, and σ is the residual standard deviation. The statistical output, along with post-hoc tests, from data of 4,672 landing manoeuvres is given in Table S5.

S1.7. Algorithm for automatic extraction of set-points of relative-rate-of-expansion

In order to automatically extract the segments of landing tracks in which bumblebees kept the relative-rate-of-expansion constant (constant-r segments), we first define methods to estimate the set-points of relative-rate-of-expansion and the variation of relative-rate-of-expansion around its estimated set-point. We later use these defined methods to construct an algorithm for automatic search of constant-r segments in each landing approach.

S1.8. Estimation of a set-point of relative-rate-of-expansion

For a track segment in which relative-rate-of-expansion is held constant, we find a zeroth-order linear regression of r to obtain the set-point (r^*) at which r is held constant (Equations S3). [align=middle]

$$r(t) = r^* + \epsilon \quad \text{(S3a)}$$

$$r^* = \frac{\sum_{i=1}^{n} r(t_i)}{n} \quad \text{(S3b)}$$

where n denotes the number of data points in that track segment and ϵ denotes the residuals. The intercept (r^*) in the linear regression is an average of the relative-rate-of-expansion observed within a constant-r segment and is used as an estimate of the set-point of r within that segment.

S1.8.1. Identification of variation around the set-points

To find the expected variation of r around the set-points in constant-r segments, we began by identifying such segments in a smaller data-set using a custom-built Graphical User Interface (GUI) in MATLAB 2020a. In this GUI, we plotted the computed state variables (V and r) against the perpendicular distance from the platform (y) and manually selected segment(s) of y (similar to the ones highlighted in red in Figure 4a) over which the instantaneous relative-rate-of-expansion (or its mean) was observed to be nearly constant. For this purpose, we use first 532 landing manoeuvres recorded for high light condition and spoke landing pattern. Out of 532 tracks, we identified 313 tracks with 355 constant-r segments (273 tracks with one segment, 38 tracks with two segments and 2 tracks with three segments) and estimated the set-point r^* using Equations S3 for each such segment.

To find the variation of r around the estimated set-points r^*, we estimated slopes and intercepts of three first-order linear fits in each such segment - one through the complete segment and two through its equal halves (Equations S4). Since, the relative-rate-of-expansion is expected to be nearly constant in the full segment and also in its two halves, the variations of three slopes, along with differences between three intercepts and the corresponding estimated set-point for the full segment, are expected to be centered around zero. We use the two halves of the segments in addition to the full segment to avoid false positives segments being detected, namely in which bumblebees are either transitioning from acceleration to deceleration phase or vice-a-versa.

$$r_{[0-1]} = m_{[0-1]}\ y_{[0-1]} + c_{[0-1]} + \epsilon \quad \text{(S4a)}$$

$$r_{[0-0.5]} = m_{[0-0.5]}\ y_{[0-0.5]} + c_{[0-0.5]} + \epsilon \tag{S4b}$$

$$r_{[0.5-1]} = m_{[0.5-1]}\ y_{[0.5-1]} + c_{[0.5-1]} + \epsilon \tag{S4c}$$

Subscripts $[0-1]$, $[0-0.5]$, and $[0.5-1]$ denote the full segment, first half, and second half of the segment, respectively, variables m and c denote the slope and intercept of a fit obtained using first-order linear regression and ϵ denotes the residuals in each case.

The distributions of three slopes ($m_{[0-1]}$, $m_{[0-0.5]}$ and $m_{[0.5-1]}$) and distributions of differences between three intercepts and the corresponding estimated set-point for the full segment ($c_{[0-1]} - r^*$, $c_{[0-0.5]} - r^*$ and $c_{[0.5-1]} - r^*$) are fitted using generalized student's t-distribution to identify their probability density functions (*tLocationScaleDistribution* in Matlab 2020a). Each such distribution is defined by a location parameter μ, scale parameter σ and shape parameter ν (σ dictates the spread of the distribution). As expected (Table S8), the location parameter (μ) for the distribution of six parameters is close to zero as the change in relative-rate-of-expansion resulting due to estimated location parameters is very small for the distances covered during constant-r segments. Moreover, the estimated scale parameters (σ) of $m_{[0-0.5]}$ and $m_{[0.5-1]}$ distributions are approximately twice the scale parameter of $m_{[0-1]}$ distribution. Similarly, the scale parameters of $c_{[0-0.5]} - r^*$ and $c_{[0.5-1]} - r^*$ distributions are approximately twice the scale parameters of $c_{[0-1]} - r^*$ distribution. Therefore, we represent the location parameters for all six distributions with the expected value zero and scale parameters of $c_{[0-1]} - r^*$, $c_{[0-0.5]} - r^*$, $c_{[0.5-1]} - r^*$, $m_{[0-1]}$, $m_{[0-0.5]}$, and $m_{[0.5-1]}$ distributions as σ_1, $2\sigma_1$, $2\sigma_1$, σ_2, $2\sigma_2$ and $2\sigma_2$, respectively (where $\sigma_1 = 0.53$ and $\sigma_2 = 4.22$). We use the represented distributions in the constant-r extraction algorithm as they are very close to the estimated ones (Figure S6).

Along with aforementioned six parameters, we define three other parameters to calculate mean acceleration in the full segment ($A_{[\text{mean},0-1]}$) and its two equal halves ($A_{[\text{mean},0-0.5]}$ and $A_{[\text{mean},0.5-1]}$), where subscripts $[\text{mean}, 0-1]$, $[\text{mean}, 0-5]$ and $[\text{mean}, 0.5-1]$ denote the mean of the acceleration computed for a full segment, first half and second half of the segment, respectively (see Materials and Methods for definition of A). As these mean acceleration values are almost always negative for the manually-identified 355 constant-r segments (Figure S6), we use these three mean acceleration parameters to identify the track segments in which a bumblebee decelerated during its landing manoeuvre.

S1.8.2. Algorithm for automatic extraction of segments with constant relative-rate-of-expansion

To automatically identify the segments of constant relative-rate-of-expansion in each landing track, we used the following algorithm:

1. Beginning with the first data point in each track, we looked at t_w time points ahead where $t_w \in \{15, 16, \ldots, 49, 50\}$ and it denotes the size of different time-windows (or segments). We chose the minimum limit for the time points (15 data points) close to the median of time points observed in 355 manually-identified constant-r segments (18 data points). The maximum limit for the time points (50 data points) is chosen as the number of data points in most manually-identified constant-r segments (353 out of 355) were below 50. Note that any long deceleration phase with more than 65 data points will be captured as two (or more) segments, but that does not affect the results observed in our study.
2. We select all those segments which satisfy the following constraints:

$$\begin{aligned}
&|c_{[0-1]} - r^*| \le f\sigma_1 \text{ and } |c_{[0-0.5]} - r^*| \le 2f\sigma_1 \text{ and } |c_{[0.5-1]} - r^*| \le 2f\sigma_1 \text{ and} \\
&|m_{[0-1]}| \le f\sigma_2 \text{ and } |m_{[0-0.5]}| \le 2f\sigma_2 \text{ and } |m_{[0.5-1]}| \le 2f\sigma_2 \text{ and} \\
&A_{[\text{mean},0-1]} \le 0 \text{ and } A_{[\text{mean},0-0.5]} \le 0 \text{ and } A_{[\text{mean},0.5-1]} \le 0
\end{aligned} \tag{S5}$$

   where f is a factor that decides the threshold on the variation allowed around the mean for first six parameters (see Section S1.8.4 for details). The first three constraints limit the variation allowed, around the constant r regression, for intercepts of first-order linear regressions in an arbitrary full segment and its two equal halves. Similarly, the next three constraints limit the variation allowed around zero for slopes of first-order linear regressions in a full segment and its two equal halves. The last three constraints helps in determining the track segments in which a bumblebee decelerates during its landing manoeuvre.
3. We repeat steps 1 and 2 for all data points in the landing track. The resulting set of selected segments contains segments that satisfy the constraint mentioned in Step 2 and may have some data points in common with other segments. Therefore, from the resulting set, we further find a subset of non-overlapping segments based on root mean square error (RMSE) in relative-rate-of-expansion. For a segment containing n data points and estimated

set-point r^*, RMSE error is calculated as given by Equation S6.

$$r_{\text{RMSE}} = \sqrt{\frac{\sum_{i=1}^{n}(r_i - r^*)^2}{n}} \tag{S6}$$

where r_i is the relative-rate-of-expansion at i-th point in a segment. After computing r_{RMSE} for all selected segments, the subset of non-overlapping segments is found by first choosing the segment with lowest r_{RMSE} and discarding all other segments with which it has data points in common. The segment with lowest r_{RMSE} in the remaining set of segments is then chosen and the segments in the remaining set it overlaps with are discarded. This process is followed until there are no segments left to choose. The set of chosen segments are then all non-overlapping and satisfy the constraints given by Equation S5. We use RMSE error for this selection process as it favours the track segments with higher number of data-points.

The resulting non-overlapping segments in a landing track are identified as the constant-r segments.

S1.8.3. Verification of results from automatic constant-r detection algorithm

To verify the results from constant-r detection algorithm, we compared the dependence of set-points of relative-rate-of-expansion (r^*) with distance from the platform (y^*) for smaller data-set (313 landing manoeuvres in which constant-r segments were manually identified) and complete data-set (4,672 landing manoeuvres for factor $f = 1$). We observed that r^* increased significantly as y^* decreased with slope (time-to-contact-rate) estimate from regression for smaller data-set ($\dot{\tau} = -0.72$) being similar to the average slope (time-to-contact-rate) estimate for complete data-set ($\dot{\tau} = -0.81$).

S1.8.4. Effect of factor f

Varying factor f has an effect similar to varying the number of standard deviations around the mean (e.g., $1-2-3$ standard deviations for $68\%-95\%-99.7\%$ empirical rule) of a normally distributed variable. Thus, increasing f leads to the detection of more false positives and fewer false negatives. Specifically, the factor f in Equation S5 determines following two aspects:

1. the threshold of the variation allowed around the mean for each parameter ($c_{[0-1]} - r^*$, $c_{[0-0.5]} - r^*$, $c_{[0.5-1]} - r^*$, $m_{[0-1]}$, $m_{[0-0.5]}$ and $m_{[0.5-1]}$) (Figure S5a)
2. the percentage of values of each parameter that lie within f scale-parameter band around the mean (Figure S5b)

Increase in f increases the threshold allowed in variation of r for a track segment to be identified as a constant-r segment, and hence, higher f can result in detection of new constant-r segments along with possible increase in the width of constant-r segments identified with lower f. However, increase in f beyond a certain value results in higher probability of detection of false positives without much increase in the true positives. We performed a sensitivity analysis by systematically varying f from $0.25 - 2.5$ and analyzing its effect on distribution of set-points of relative-rate-of-expansion (r^*) identified, r^* dynamics with distance to the platform (y^*) and effect of environmental conditions on this dynamics (Figure S5c,d). We observed that all of these results remain essentially unaltered in the tested wide-range of f.

S1.9. Calculation of relative approach speeds of the hybrid landing strategy

We tested how the here-described hybrid landing strategy compares to both the constant-r strategy and the constant-$\dot{\tau}$ strategy. For this, we calculated for each set of two consecutive constant-r segments, the hybrid-to-constant-r speed ratio and the hybrid-to-constant-$\dot{\tau}$ speed ratio as U_H/U_r and $U_H/U_{\dot{\tau}}$, respectively. Here, U_H is the average flight speed during the set of two consecutive constant-r segments, defined as $U_H = \Delta y_2/\Delta t_2$, where Δy_2 and Δt_2 are the distance travelled and flight duration, respectively (Figure 4a in the main text). U_r is the equivalent flight speed if the bumblebee would have used the constant-r strategy and continued to fly at its first set-point (r_1^*), and is defined as $U_r = 2/(r_1^*(y_{start} + y_{end}))$, where y_{start} and y_{end} are the distances from the platform at the start and end of the flight section. $U_{\dot{\tau}}$ is the equivalent speed if the bumblebee would have used the constant-$\dot{\tau}$ strategy to fly continuously at the average time-to-contact-rate observed in our data-set ($\dot{\tau} = -0.87$), with an initial approach speed equal to that at the start of a flight section. The resulting average speed $U_{\dot{\tau}}$ is then calculated as the average of approach velocities computed using Equation S8a. For this purpose, we used 1008 instead of 1015 landing manoeuvres with two constant-r segments because remaining seven landing manoeuvres corresponded to bumblebees flying away from the platforms in between the two consecutive constant-r segments.

S1.10. Governing equations for a constant time-to-contact-rate landing strategy

For an animal approaching a platform (Figure 1a), at time t, we denote its distance to the platform as $y(t)$, approach velocity as $V(t)$, relative-rate-of-expansion as $r(t)$, time-to-contact parameter as $\tau(t)$, time-to-contact-rate parameter as $\dot{\tau}(t)$ and following holds:

$$V(t) = -\frac{dy(t)}{dt}, \; r(t) = \frac{1}{\tau(t)} = \frac{V(t)}{y(t)} \text{ and } \dot{\tau}(t) = \frac{d\tau(t)}{dt} \tag{S7}$$

The equations that govern dependence of state variables r and V on y for a constant $\dot{\tau}$ landing strategy are derived elsewhere Baird, Boeddeker, Ibbotson and Srinivasan (2013) and only final equations are depicted here (Equations S8).

$$V(t) = c_1 \; y(t)^{\dot{\tau}+1} \tag{S8a}$$

$$r(t) = c_2 \; y(t)^{\dot{\tau}} \tag{S8b}$$

where c_1 and c_2 are constants that depend on initial conditions of the state variables y, V and r.

S1.11. Parametrization of probability density functions used in this study

We use two probability density functions (pdf) in this study which are parametrized as follows:

1. Gamma distribution (*GammaDistribution* in MATLAB 2020a) - For a shape parameter a and scale parameter b, the pdf of the gamma distribution is

$$p(x \mid a, b) = \frac{1}{b^a \Gamma(a)} x^{a-1} e^{\frac{-x}{b}} \tag{S9}$$

where $\Gamma(\cdot)$ is the Gamma function.

2. Generalized student's t-distribution (*tLocationScaleDistribution* in MATLAB 2020a) - For a location parameter μ, scale parameter σ and shape parameter ν, the pdf of generalized student's t-distribution is

$$p(x \mid \mu, \sigma, \nu) = \frac{\Gamma\left(\frac{\nu+1}{2}\right)}{\sigma\sqrt{\nu\pi}\,\Gamma\left(\frac{\nu}{2}\right)} \left[\frac{\nu + \left(\frac{x-\mu}{\sigma}\right)^2}{\nu}\right]^{-\left(\frac{\nu+1}{2}\right)} \tag{S10}$$

where $\Gamma(\cdot)$ is the Gamma function.

References

Baird, E., Boeddeker, N., Ibbotson, M.R., Srinivasan, M.V., 2013. A universal strategy for visually guided landing. Proceedings of the National Academy of Sciences 110, 18686–18691. doi:10.1073/pnas.1314311110.

Chang, J.J., Crall, J.D., Combes, S.A., 2016. Wind alters landing dynamics in bumblebees. Journal of Experimental Biology 219, 2819–2822. doi:10.1242/jeb.137976.

Straw, A.D., Branson, K., Neumann, T.R., Dickinson, M.H., 2011. Multi-camera Real-time Three-dimensional Tracking of Multiple Flying Animals. Journal of The Royal Society Interface 8, 395–409. doi:10.1098/rsif.2010.0230.

Svoboda, T., Martinec, D., Pajdla, T., 2005. A convenient multicamera self-calibration for virtual environments. Presence 14, 407–422. doi:10.1162/105474605774785325.
